# Salmonella Facilitates Iron Acquisition through UMPylation of Ferric Uptake Regulator

**DOI:** 10.1128/mbio.00207-22

**Published:** 2022-05-09

**Authors:** Haihong Jia, Nannan Song, Yue Ma, Fengyu Zhang, Yingying Yue, Weiwei Wang, Cuiling Li, Hui Li, Qi Wang, Lichuan Gu, Bingqing Li

**Affiliations:** a Department of Clinical Laboratory, Shandong Provincial Hospital Affiliated to Shandong First Medical University, Jinan, China; b Department of Pathogen Biology, School of Basic Medicine, Shandong First Medical University & Shandong Academy of Medical Sciences, Jinan, China; c State Key Laboratory of Microbial Technology, School of Life Sciences, Shandong University, Qingdao, China; d Key Lab for Biotech-Drugs of National Health Commission, Jinan, China; e Key Lab for Rare & Uncommon Diseases of Shandong Province, Jinan, China; New York University School of Medicine

**Keywords:** Fur, *Salmonella*, UMPylation, YdiU, iron metabolism

## Abstract

Iron limitation is a universal strategy of host immunity during bacterial infection. However, the mechanisms by which pathogens antagonize host nutritional immunity have not been fully elucidated. Here, we identified a requirement for the UMPylator YdiU for this process in Salmonella. The expression of YdiU was dramatically induced by the metal starvation signal. The intracellular iron content was much lower in the Δ*ydiU* strain than in wild-type Salmonella, and the Δ*ydiU* strain exhibited severe growth defect under metal deficiency environments. Genome-wide expression analyses revealed significantly decreased expression of iron uptake genes in Δ*ydiU* strain compared with the wild-type strain. Interestingly, YdiU did not affect the expression level of the major iron uptake regulator Fur but directly UMPylated Fur on its H118 residue *in vivo* and *in vitro*. UMPylation destroyed the Fur dimer, promoted Fur aggregation, and eliminated the DNA-binding activity of Fur, thus abolishing the ability of Fur to inhibit iron uptake. Restricting Fur to the deUMPylated state dramatically eliminates Salmonella iron uptake in iron deficiency environments. In parallel, YdiU facilitates Salmonella survival within host cells by regulating the iron uptake pathway.

## INTRODUCTION

Iron is an essential element for almost all living organisms. As a cofactor, the iron ion participates in multiple important life processes in bacteria, including nitrogen fixation, DNA synthesis, and damage repair (1, 2). Although iron is abundant in nature, the direct utilization of iron by organisms is difficult because of its poor solubility (3, 4). To prevent pathogenic bacteria from acquiring iron, host cells employ multiple strategies to limit the iron that is available for microorganisms in a process known as nutritional immunity (5–7).

To counter host nutritional immunity, bacteria have evolved multiple approaches to acquire sufficient iron from their hosts and other iron-deficient environments. The synthesis of enterobactin (Ent), a catechol siderophore with high iron affinity, is a main survival strategy in *Enterobacteriaceae* species, including Escherichia coli, Salmonella enterica serovar Typhimurium, and Klebsiella pneumonia under iron-deficient conditions (8–11). Ent is assembled by the nonribosomal peptide synthetase (NRPS) composed of EntB, EntE, and EntF proteins (12). Synthesized Ents are secreted out of the cell to capture iron atoms. Fe-bound Ents are then recognized and transported into the cytoplasm by a series of ferric-enterobactin uptake proteins (FepABCDEG) (13). Intriguingly, although iron is necessary, it is harmful to bacteria at high concentration. The Fenton reaction will occur in the presence of a high concentration of intracellular iron and H_2_O_2_, producing a large number of hydroxyl radicals that subsequently lead to macromolecular damage and bacterial death (14). Thus, iron is required to be at an appropriate level so bacteria can obtain needed iron but also avoid excessive iron-induced damage.

The highly conserved gene *fur* (ferric uptake regulator) is the major regulator of iron homeostasis in bacteria (15–17). When iron ions are abundant, Fe-Fur binds the promoters of iron uptake-related genes, interfering with RNA polymerase binding to block transcription of these genes (18, 19). When bacteria move from an iron-rich environment to an iron-deficient environment (such as inside host cells), the transcription of iron uptake genes can restart (20–22). Lee et al. proposed a Fur working model in which a small amount of intracellular iron results in the release of iron ions from Fur and Fur free from Fe^2+^ releases from the DNA, allowing RNA polymerase to bind the promoters and transcribe the iron uptake (18). However, there are uncertainties in this widely accepted mechanism. For example, in Campylobacter jejuni, Fur forms dimers and binds the promoter regions of target genes even in the absence of the iron cofactor (23). In addition, metal-unbound mutants of Bradyrhizobium japonicum Fur still repressed gene expression *in vivo* (24). More importantly, *in vitro* studies have shown that in addition to iron, Fur can bind manganese and cobalt ions to achieve high DNA binding activity (18, 25), suggesting that Fur regulation is more complex than strict iron dependence. A protein named EIIA was reported to directly interact with Fur and regulate its DNA binding activity (26). Recently, we reported that YdiV, the flagellar regulatory protein, can change the conformation of Fur with the assistance of the chaperone SlyD during the folding of Fur, interfering with its DNA binding activity (27).

Protein posttranslational modification is an important way to regulate protein function, but modification of Fur has not been reported. We previously identified a ubiquitous and highly conserved protein, YdiU, as an enzyme that catalyzes UMPylation, modifying bacterial proteins with UMP (28). Mass spectrometry-based proteomic analysis identified 46 UMPylated bacterial proteins in the YdiU-expressing Salmonella strain, including Fur (28). However, the function of YdiU after induction during iron deficiency and the potential effects of YdiU on Fur remain unclear.

Previous studies suggested a relationship between YdiU and bacterial iron metabolism. First, the expression of YdiU was reported to be regulated by an iron signal-related transcription factor, IcsR (29, 30). In an *iscA sufA* mutant strain lacking iron-sulfur clusters and known iron uptake-related genes, the expression of YdiU is dramatically upregulated 8.3-fold (31). Domain analysis of the genomes from multiple species revealed that *fur* and *ydiU* genes are located in close proximity, increasing the probability of coordinated expression and suggesting an association between YdiU and Fur (32). Given that Fur can be UMPylated in YdiU-expressing Salmonella, we proposed that YdiU can modify Fur via UMPylation to regulate iron uptake in bacteria when iron was limited.

The experiments performed in this study support the above-described hypothesis. First, we confirmed that YdiU could be effectively induced by metal deficiency. Next, we found that, compared with the wild-type strain, the *ydiU* mutant strain exhibited significantly reduced viability and significantly decreased intracellular content of iron under metal-deficient conditions. Proteomics data revealed that expression of genes related to Ent biogenesis and acquisition significantly decreased in the Δ*ydiU* cells with no obvious difference in Fur expression levels, indicating that the regulation of iron uptake-related genes by YdiU occurred after translation of Fur. More importantly, we found that YdiU catalyzed UMPylation of Fur both *in vivo* and *in vitro*. UMPylation destroyed Fur dimers and made Fur more likely to aggregate. Moreover, the UMPylated Fur lost its DNA binding activity. The regulation of iron uptake by YdiU was independent of its upstream gene *ydiV* but dependent on *fur* and UMPylation activity. The results of *in vivo* experiments indicated that the YdiU-mediated regulation of iron uptake is critical for Salmonella survival for cells experiencing iron deficiency. Overall, we revealed a mechanism by which posttranslational modification can regulate Fur function and effectively initiate bacterial iron uptake, facilitating Salmonella survival within host cells.

## RESULTS

### YdiU is involved in the iron metabolism pathway of Salmonella.

**FIG 1:**
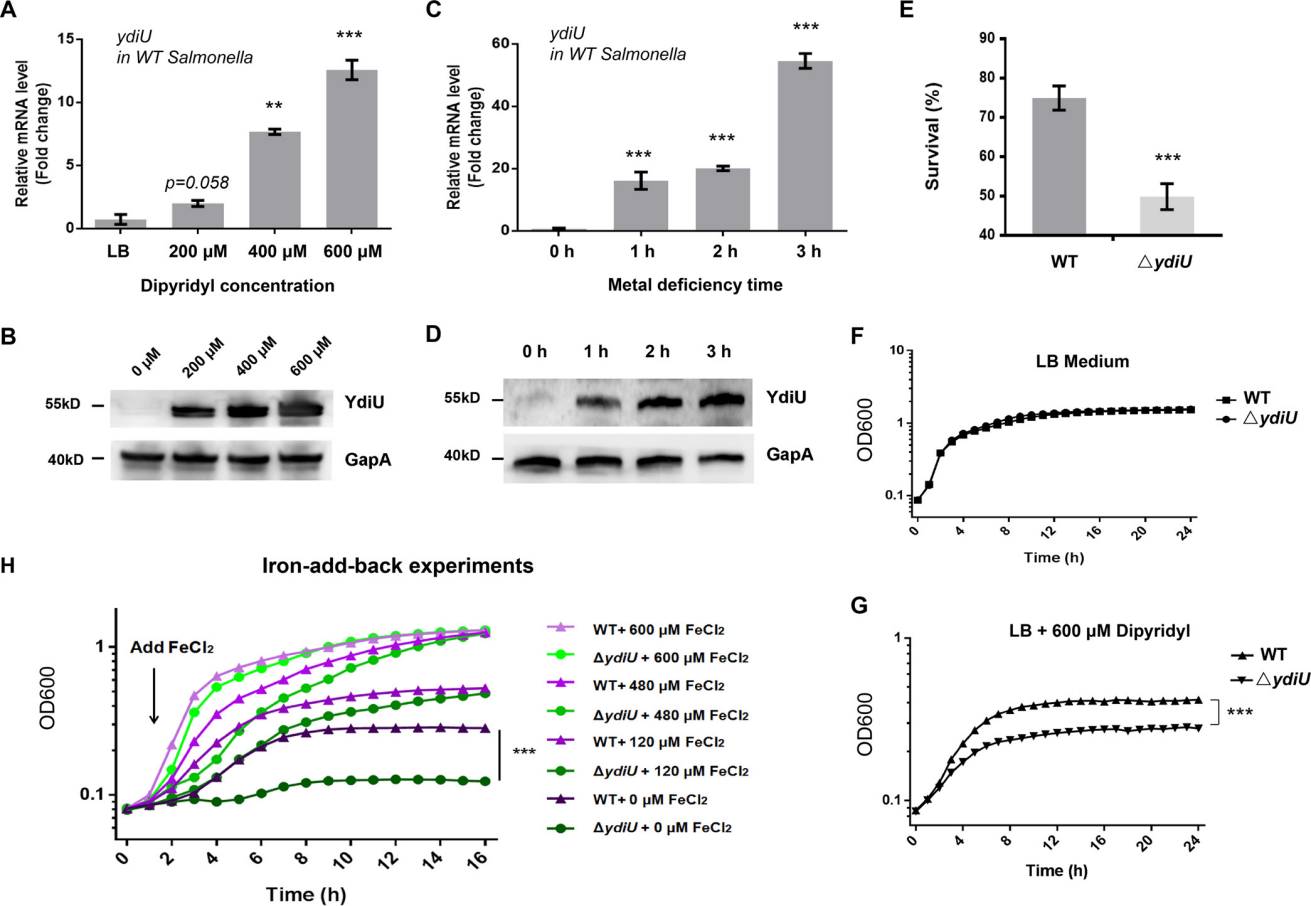
YdiU is involved in the iron metabolism pathway. (A and B) The transcription and protein levels of YdiU in Salmonella cultivated with different concentrations of 2,2′-dipyridyl were detected with qRT-PCR and Western blotting, respectively. GapA (also known as glyceraldehyde-3-phosphate dehydrogenase [GAPDH]) was used as a loading control. (C and D) The transcription and protein levels of YdiU in Salmonella treated with 600 μM 2,2′-dipyridyl for the indicated times were detected with qRT-PCR and Western blotting, respectively. (E) The survival rates of WT and Δ*ydiU* strains after metal deprivation for 3 h. (F and G) The growth curves of WT and Δ*ydiU* cultivated in LB medium or metal-limited medium. (H) Iron add-back experiments of wild-type and Δ*ydiU* cells. Different concentration of FeCl_2_ was added into the corresponding cultures 2 h after metal ion deprivation. The above-described experiments were performed as three replicates, and the mean values are presented. ***, *P* < 0.001; **, *P* < 0.01; *, *P* < 0.05 compared with the WT strain cultivated in LB medium (A and C) or with the WT strain under identical conditions (E).

Our previous data showed that YdiU was expressed at a very low level when Salmonella was cultured in Luria-Bertani (LB) medium but strongly induced when metal-chelating agent 2,2′-dipyridyl was added (28). Dipyridyl is a metal chelator that binds nickel, cobalt, iron, and zinc with almost equal affinity. To further investigate the correlation between metal starvation and YdiU expression, the mRNA and protein levels of YdiU were measured for Salmonella cultured in LB medium supplemented with different concentrations of dipyridyl. Our data showed that the expression of YdiU was efficiently induced by dipyridyl, and expression increased with increased dipyridyl concentration (Fig. 1A and B). The real-time expression of YdiU was determined 1 h, 2 h, or 3 h after the addition of dipyridyl. The results indicated that YdiU was expressed 1 h after metal starvation and continued to increase with increased time (Fig. 1C and D). Specifically, when cells were incubated with dipyridyl for 1, 2, and 3 h, the mRNA level of *ydiU* increased 16.2-, 19.8-, and 54.6-fold, respectively (Fig. 1C), and protein levels of YdiU increased 14.5-, 23.7-, and 44.2-fold, respectively (Fig. 1D). These data demonstrate that the metal starvation signal dramatically induced YdiU expression.

To explore the function of YdiU in metal homeostasis, cell survival was monitored using wild-type (WT) and Δ*ydiU* Salmonella grown under metal-limited conditions. The survival rate of WT cells was 74.94% in metal-deprived medium, but the survival rate of the Δ*ydiU* strain was only 49.78% under the same conditions (Fig. 1E). Importantly, although the growth curves of WT and Δ*ydiU* strains were similar in LB medium, Δ*ydiU* cells cultured in metal-deprived medium grew significantly differently than WT cells, with the optical density at 600 nm (OD_600_) value of WT cells reaching a plateau of about 0.41 but Δ*ydiU* cells reaching only 0.25 (Fig. 1F and G). The metal add-back experiments then were performed. The result showed that the addition of iron rescues the growth defect of Δ*ydiU* Salmonella under metal deficiency conditions in a dose-dependent manner (Fig. 1H). Iron rescues growth defect, but an influence of other metals cannot be fully excluded.

### YdiU facilitates iron absorption by activating iron uptake gene transcription.

**FIG 2:**
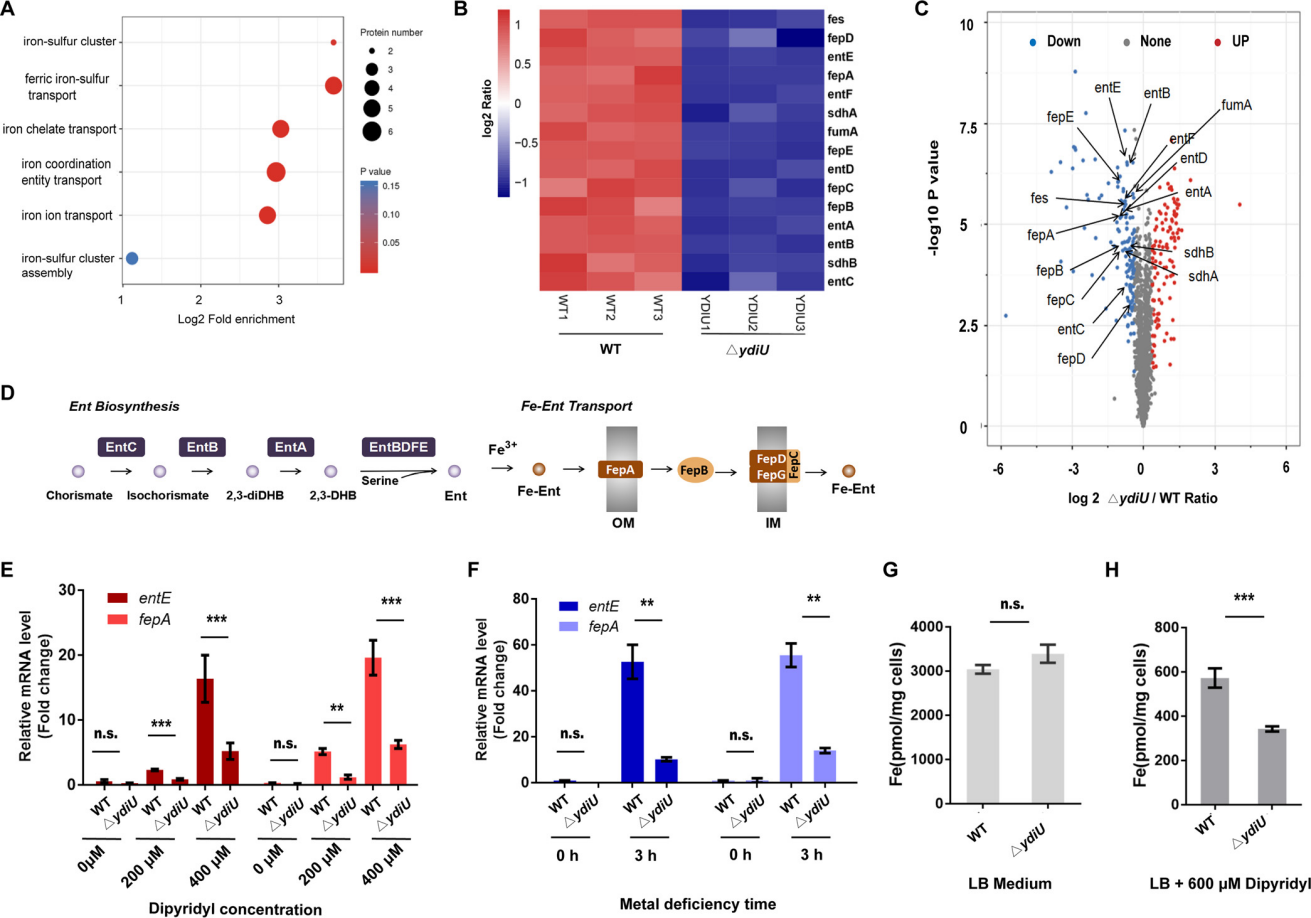
YdiU facilitates iron absorption by activating iron uptake genes. (A to C) Differentially expressed proteins of WT and Δ*ydiU* strains under metal-limited conditions were analyzed by LC-MS/MS-based proteomics. (A) Pathway enrichment analysis highlighted proteins involved in enterobactin biosynthesis and uptake. (B) Heatmap of the fold changes of enterobactin biosynthesis and uptake proteins. (C) Volcano plots representing the proteomic results. The blue and red spots represent the downregulated and upregulated proteins, respectively. Iron-related proteins are labeled. (D) A summary of enterobactin biosynthesis and uptake pathway. (E and F) The expression levels of *entE* and *fepA* in WT and Δ*ydiU* strains under normal or metal-limited conditions were separately measured by qRT-PCR. GADPH was used as an internal reference. (G and H) The intracellular iron content of WT and Δ*ydiU* strains in LB (G) or metal-limited (H) medium was monitored by ICP-MS. The above-described experiments were performed as three replicates, and the mean values are presented. ***, *P* < 0.001; **, *P* < 0.01; *, *P* < 0.05; n.s., *P* > 0.05.

To further clarify the function of YdiU, mass spectrometry-based proteomics were used to analyze the differences in global protein expression in WT and Δ*ydiU* cells grown with dipyridyl (see Fig. S1 in the supplemental material). Overall, a total of 251 proteins were found to be differentially expressed when the *ydiU* gene was deleted, with fold change of >30% and *P* value of <0.01, of which 113 genes were upregulated and 138 genes were downregulated. These proteins fall into several categories, including cell motility, iron homeostasis, virulence, and energy production. Because our focus is on the function of YdiU on iron metabolism, we next analyzed genes related to iron metabolism. Interestingly, several iron uptake-related proteins were significantly repressed in Δ*ydiU* strain (Fig. 2A to C). Remarkably, the enterobactin biosynthesis proteins (EntABCDEF) and ferric-enterobactin uptake proteins (FepABCDEG) were all reduced to ~50% in Δ*ydiU* strain compared with the WT strain. Synthesis of enterobactin is a major strategy that Salmonella uses to salvage iron within host cells and under other iron-limited conditions (Fig. 2D) (9, 33, 34), so we hypothesized that YdiU facilitates iron absorption by increasing the levels of proteins involved in enterobactin biosynthesis and ferric-enterobactin uptake. The altered expression of iron uptake genes was confirmed using quantitative PCR (qPCR) by representative genes *entE* and *fepA*. The expression levels of *entE* and *fepA* in the Δ*ydiU* strain dropped to 20% to 50% of the levels in the WT strain under metal-deficient conditions (Fig. 2E and F). The complementation of *ydiU* in the Δ*ydiU* strain restores the expression levels of these genes, confirming this effect is due to the requirement of YdiU (Fig. S2). Compared with the Δ*ydiU* strain, the transcription of *entE* and *fepA* genes in the p*ydiU* strain showed dramatic increases under metal-deficient conditions, 21.44-fold for *entE* and 3.5-fold for *fepA* (Fig. S2B and C). To further investigate the role of YdiU in iron absorption, we measured the intracellular iron concentrations in the WT and Δ*ydiU* strains by inductively coupled plasma mass spectrometry (ICP-MS) in LB and metal-deficient medium. Interestingly, there was no significant difference in the cellular iron concentration in the WT and Δ*ydiU* strains under iron-rich conditions (Fig. 2G). However, in metal-deficient medium, the intracellular iron concentration of Δ*ydiU* strain was significantly reduced to ~60% of that in the WT strain, with an average of 572.7 pmol/mg cells in the WT strain and 340.1 pmol/mg cells in *ydiU* mutant strains (Fig. 2H). These data demonstrate that YdiU acts as an activator for iron uptake by upregulating the transcription of iron uptake genes.

10.1128/mbio.00207-22.1 FIG S1 Quantitative proteomic identifies Salmonella YdiU as a global regulator under iron deficiency conditions. (A) Total proteomic data determined by LC-MS/MS were assessed by principle-component analysis. (B) Differentially expressed proteins with fold change of >30% and *P* value of <0.01 in WT and Δ*ydiU* strains, with 113 upregulated and 138 downregulated. (C) Volcano plot of detected proteins by LC-MS/MS. (D and E) Pathway enrichment analysis of differentially expressed protein performed using GO analysis (D) and KEGG analysis (E). Download FIG S1, TIF file, 1.9 MB. Copyright © 2022 Jia et al. 2022 Jia et al. https://creativecommons.org/licenses/by/4.0/ This content is distributed under the terms of the Creative Commons Attribution 4.0 International license.

10.1128/mbio.00207-22.2 FIG S2 Complementation of YdiU into Δ*ydiU* strain fully rescued expression of iron uptake genes. The transcription levels of *ydiU* (A), *entE* (B), and *fepA* (C) in WT, Δ*ydiU*, and Δ*ydiU* p*ydiU* strains under metal-limited conditions were detected by qRT-PCR. Experiments were performed as three replicates, and the mean values are presented. ***, *P* < 0.001; **, *P* < 0.01; *, *P* < 0.05. Download FIG S2, TIF file, 1.0 MB. Copyright © 2022 Jia et al. 2022 Jia et al. https://creativecommons.org/licenses/by/4.0/ This content is distributed under the terms of the Creative Commons Attribution 4.0 International license.

### Neither *fur* mRNA nor Fur protein level is affected by YdiU.

The iron uptake pathway is mainly controlled by Fur in Salmonella (35). Previous transcriptomics data demonstrated that the expression of the *fur* gene is at a high level when Salmonella organisms enter host cells, and expression of iron uptake genes increases 10- to 20-fold after Salmonella organisms enter host cells (36, 37). The strategy by which Salmonella can relieve the inhibitory effect of Fur to activate iron uptake genes has not been fully elucidated. Our proteomic data revealed no obvious difference at the protein level of Fur between WT and Δ*ydiU* strains under metal-deficient conditions (Fig. S3A). Next, qRT-PCR was used to evaluate a potential effect of YdiU on *fur* transcription (Fig. S3B and C). The results clearly showed that the mRNA level of *fur* did not significantly change with the expression of YdiU during iron deficiency. These data indicate that the regulation by YdiU of the iron uptake pathway must occur at a step after *fur* transcription.

10.1128/mbio.00207-22.3 FIG S3 YdiU exhibits no significant effect on the expression of Fur. (A) Fold changes in protein levels of Fur monitored from LC-MS/MS based proteomics. (B) Relative mRNA levels of Fur in WT and ΔYdiU strains cultured under LB medium and metal-limited medium were detected by qRT-PCR. (C) Relative mRNA levels of *fur* in WT, Δ*ydiU*, and p*ydiU* p*ydiU* strains under metal-limited condition. Experiments were performed as three replicates, and the mean values are presented. The statistical significance is indicated. Download FIG S3, TIF file, 1.2 MB. Copyright © 2022 Jia et al. 2022 Jia et al. https://creativecommons.org/licenses/by/4.0/ This content is distributed under the terms of the Creative Commons Attribution 4.0 International license.

### YdiU UMPylates Fur both *in vivo* and *in vitro*.

**FIG 3:**
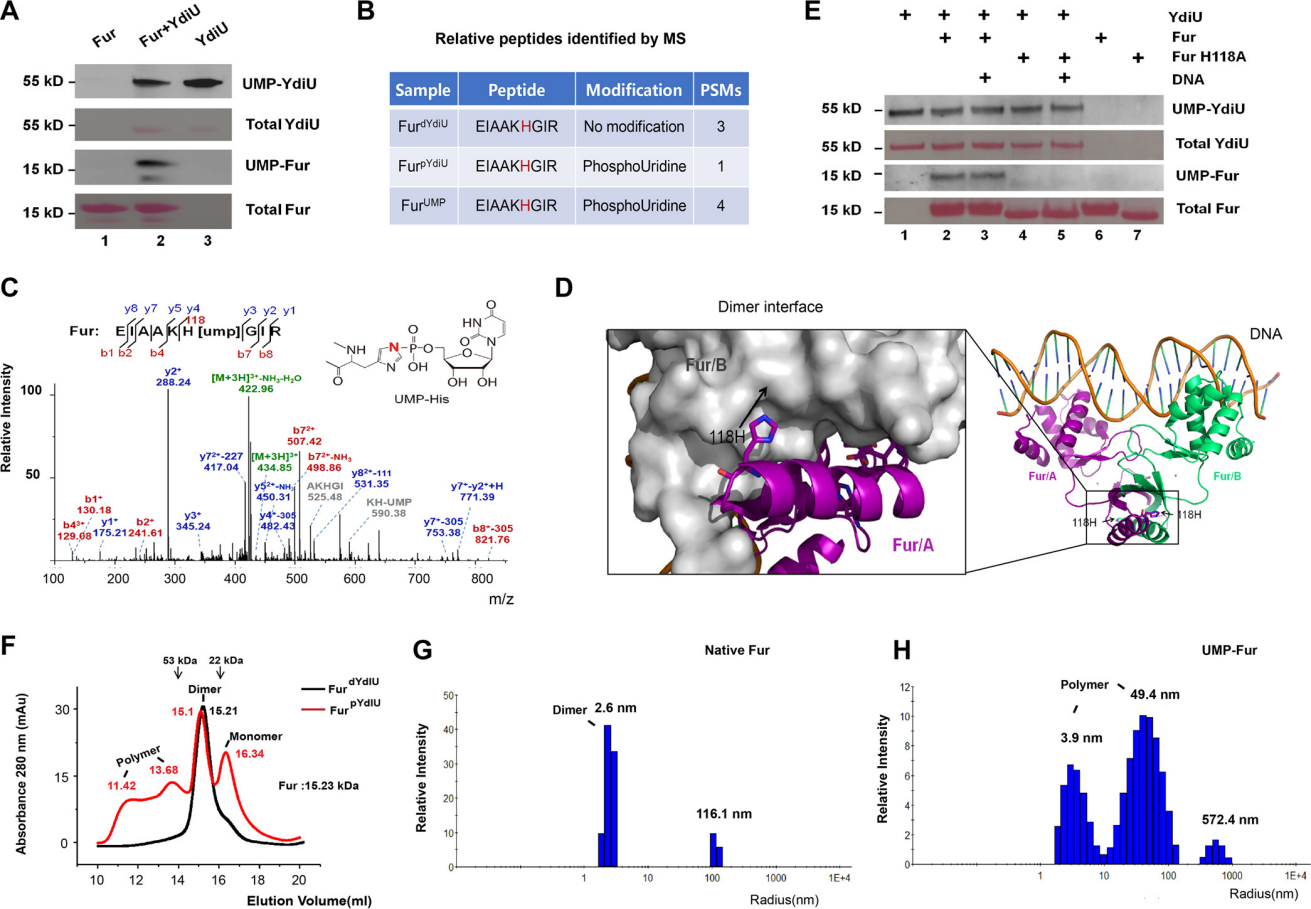
UMPylation of Fur by YdiU at H118 alters Fur’s aggregation state. (A) *In vitro* UMPylation of Fur catalyzed by YdiU. UMPylation reaction mixtures were performed with biotin-16-UTP and MnCl_2_, followed by avidin blotting. Total proteins were visualized by Ponceau S staining. (B) UMPylated peptides of Fur identified by mass spectrometry in Fur^dYdiU^, Fur^pYdiU^, and Fur^UMP^. (C) Electrospray ionization tandem mass spectrometry (MS/MS) spectra of UMPylated peptides identified in Fur^UMP^. The b and y ions are indicated along the peptide sequence above the spectra. Unique ions (111.1, 227.1, and 305.1 Da) corresponding to the neutral loss of the UMP group were detected in the MS/MS spectra of UMPylated peptides. (D) Structural presentation shows that H118 is located in the dimeric interface. H118 is highlighted in stick form. Shown is PDB entry 4RB3. (E) *In vitro* UMPylation of native Fur and FurH118A in the absence and the presence of excess DNA. The UMPylation reactions were performed with biotin-16-UTP and MnCl_2_, followed by avidin blotting. Total proteins were visualized by Ponceau S staining. (F) Size-exclusion chromatography (SEC) results of Fur^dYdiU^ and Fur^pYdiU^. The elution volume of each peak value is indicated in the corresponding colors. (G and H) The characteristics of native Fur and UMPylated Fur were determined by dynamic light scattering. Both YdiU and Fur proteins used in the above-described experiments were from E. coli.

YdiU acts as an enzyme to catalyze posttranslational modification of protein (28, 38). Previous mass spectrometry-based proteomic analysis uncovered 46 UMPylated proteins in the YdiU-expressing Salmonella strain (28). Remarkably, Fur is one of these substrates and was found to be UMPylated on its H33 residue (Fig. S4A). Fur is the major regulator of iron uptake, which suggests that YdiU performs its function by directly UMPylating Fur. Fur and YdiU proteins are highly conserved in Salmonella and E. coli, and since the expression and solubility of the Salmonella protein is often lower than that of the E. coli protein when expressed in E. coli, we used the E. coli homologous proteins (Fur and YdiU) for *in vitro* validation. We purified *E. coli* YdiU and Fur proteins from *ydiU* knockout E. coli (Fur^dYdiU^) and YdiU-expressing *E. coli* (Fur^pYdiU^), respectively (Fig. S5), and then performed *in vitro* UMPylation experiments using these purified proteins. The results clearly showed that YdiU could catalyze *in vitro* UMPylation of Fur (Fig. 3A). To ascertain the site(s) of UMPylation, we used mass spectrometry to analyze Fur^dYdiU^, Fur^pYdiU^, and Fur after *in vitro* UMPylation by YdiU (Fur^UMP^). Interestingly, UMPylated H33 was not detected, but H118 was identified as a novel UMPylation site. Further, UMPylation on Fur H118 was detected in Fur^pYdiU^ and Fur^UMP^ but not in Fur^dYdiU^, demonstrating H118 acts as an UMPylation site both *in vitro* and *in vivo* (Fig. 3B and C).

10.1128/mbio.00207-22.4 FIG S4 H33 of Fur is UMPylated in YdiU-expressing Salmonella. (A) Electrospray ionization tandem mass spectrometry (MS/MS) spectra of UMPylated peptides on H33 identified in YdiU-expressing Salmonella. Fur protein was from *S.* Typhimurium. (B) Structural superposition of apo-Fur and holo-Fur (Mn^2+^-bound). The overall structures are shown in cartoon form, and H33 is highlighted by sticks. The displacement of H33 between apo-Fur and holo-Fur is indicated. Shown are PDB entries 4RAY and 4RAZ. Download FIG S4, TIF file, 2.0 MB. Copyright © 2022 Jia et al. 2022 Jia et al. https://creativecommons.org/licenses/by/4.0/ This content is distributed under the terms of the Creative Commons Attribution 4.0 International license.

10.1128/mbio.00207-22.5 FIG S5 Purification results of YdiU, Fur^dYdiU^, and Fur^pYdiU^. Protein names are marked on the top of the picture. C, whole cell; S, supernatant; F, flowthrough sample after Ni-NTA affinity column; E, purified protein after Ni-NTA affinity column. Both YdiU and Fur used in these experiments were from *E. coli*. Download FIG S5, TIF file, 2.0 MB. Copyright © 2022 Jia et al. 2022 Jia et al. https://creativecommons.org/licenses/by/4.0/ This content is distributed under the terms of the Creative Commons Attribution 4.0 International license.

### The UMPylated sites are located in positions of Fur essential for dimerization and iron binding.

To investigate the effect of UMPylation on Fur, we analyzed the spatial structure of UMPylation sites H33 and H118. H33 is located near the iron-binding site (Fig. S4B). In the apo-Fur structure, H33 is relatively exposed, but in holo-Fur, H33 is coordinated with manganese (19). Thus, UMPylation of Fur by YdiU might be regulated by the binding state of Fur, where UMPylation of H33 might be favored for the iron-unbound form of Fur. Residue H118 was also detected as a site of UMPylation both *in vivo* and *in vitro* (Fig. 3B and C), and this residue is located on the surface of the Fur homodimer (Fig. 3D). The addition of UMP moiety to H118 could disrupt the dimer interface and thereby alter the protein aggregation state of Fur. By sequence alignment, we found that these UMPylated residues (H33 and H118) are highly conserved across Fur’s orthologs, suggesting the regulation of Fur by YdiU-mediated UMPylation is employed widely (Fig. S6). The H118A mutant of Fur could not be UMPylated by YdiU, confirming H118 was the major UMPylated site of Fur *in vitro* (Fig. 3E).

10.1128/mbio.00207-22.6 FIG S6 UMPylated residues (H33 and H118) are highly conserved across Fur orthologs. CLUSTALW alignment between Salmonella Fur, E. coli Fur, and Fur from other bacteria. GenBank accessio nno. P0A9A9, *E. coli* Fur; Q83S79, Shigella flexneri Fur; Q7CQY3, Salmonella Typhimurium Fur; D2TNJ5, Citrobacter rodentium Fur; A0A0H3CN37, Enterobacter cloacae subsp. *cloacae* Fur; C9XZ74, Cronobacter turicensis Fur; P45599, Klebsiella pneumoniae Fur; B2VBQ6, Erwinia tasmaniensis Fur; P33086, Yersinia pestis Fur. Download FIG S6, TIF file, 1.4 MB. Copyright © 2022 Jia et al. 2022 Jia et al. https://creativecommons.org/licenses/by/4.0/ This content is distributed under the terms of the Creative Commons Attribution 4.0 International license.

### The UMPylated Fur presents a different aggregation state with native Fur.

To further investigate the effect of UMPylation on the characteristics of Fur, we analyzed Fur^dYdiU^ and Fur^pYdiU^ using size exclusion chromatography (Fig. 3F). Fur^dYdiU^ exhibited a single elution peak at 15.21 mL, implying that Fur forms a homodimer without UMPylation. Interestingly, Fur^pYdiU^ showed a more complex result, with four major elution peaks (11.42 mL, 13.68 mL, 15.1 mL, and 16.34 mL), demonstrating a changed aggregation state of Fur after YdiU-mediated UMPylation. The elution peak at 16.34 mL shows that UMPylation split the Fur dimer into the Fur monomer form. The peaks at 11.42 mL and 13.68 mL suggest that UMPylation converts Fur to an aggregation-prone state. *In vitro* UMPylation was performed using Fur^dYdiU^ and YdiU to obtain fully UMPylated Fur (Fur^UMP^), and the characteristics of native Fur and Fur^UMP^ were compared using native gel and dynamic light scattering (DLS). The bands of Fur^UMP^ are shifted compared with those of native Fur (Fig. S7). Large polymers with an ~49.4-nm radius were produced after UMPylation, as detected by dynamic light scattering (Fig. 3G and H). All these results showed that Fur became less stable and more aggregated after UMPylation.

10.1128/mbio.00207-22.7 FIG S7 Native gel analysis of UMPylated and native Fur proteins. Native polyacrylamide gel electrophoresis experiments were carried out with a native gel containing 12% acrylamide. Samples of 5 μg UMPylated and native Fur proteins were electrophoretically separated in buffer with or without 10 mM MnCl_2_. Fur used in this experiment was from E. coli. Download FIG S7, TIF file, 2.1 MB. Copyright © 2022 Jia et al. 2022 Jia et al. https://creativecommons.org/licenses/by/4.0/ This content is distributed under the terms of the Creative Commons Attribution 4.0 International license.

### UMPylation impaired the DNA-binding activity of Fur.

**FIG 4:**
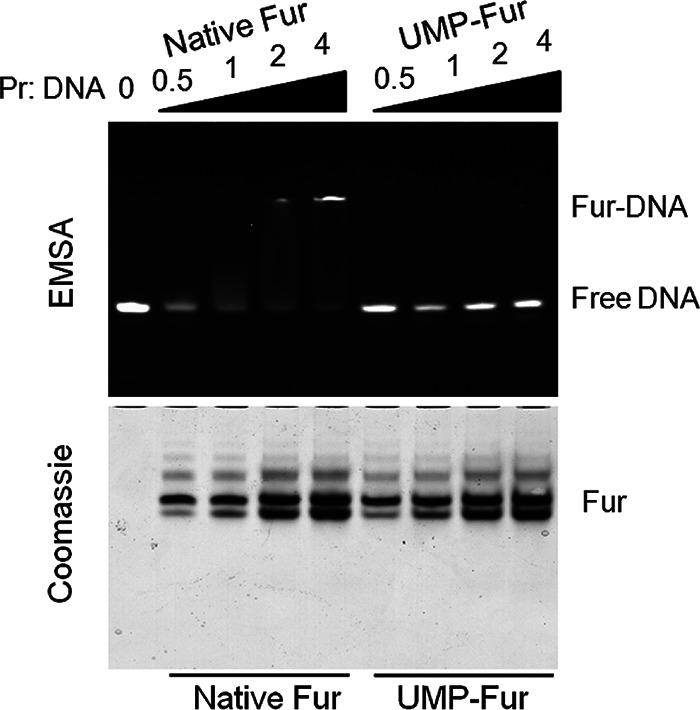
UMPylation prevents Fur from binding target DNA. Electrophoretic mobility shift assays (EMSAs) were performed for native Fur and UMPylated Fur with Fur box DNA. In these assays, 10 nmol FAM-labeled Fur box DNA was mixed with 0 to 40 nmol native Fur or UMPylated Fur for 10 min at 30°C and then analyzed by EMSA. The upper and lower pictures show the same gel imaged by fluorescence or stained with Coomassie brilliant blue. The experiment was repeated three times with similar results. Fur used in the above-described experiments was from E. coli.

Fur inhibits the transcription of iron uptake genes by directly interacting with promoter DNA (39, 40). The DNA binding ability of native Fur and UMPylated Fur was compared using electrophoretic mobility shift assay (EMSA) and using different molar ratios of Fur and DNA. A clear DNA shift was detected for a molar ratio of native Fur and DNA greater than 1; however, no shift was detected using UMPylated Fur even at a ratio of protein and DNA greater than 4 (Fig. 4). The complete loss of DNA binding activity by UMPylated Fur suggests that YdiU regulates the iron uptake pathway by UMPylating and inactivating Fur.

### Regulation of iron uptake by YdiU depended on Fur and UMPylation activity.

**FIG 5:**
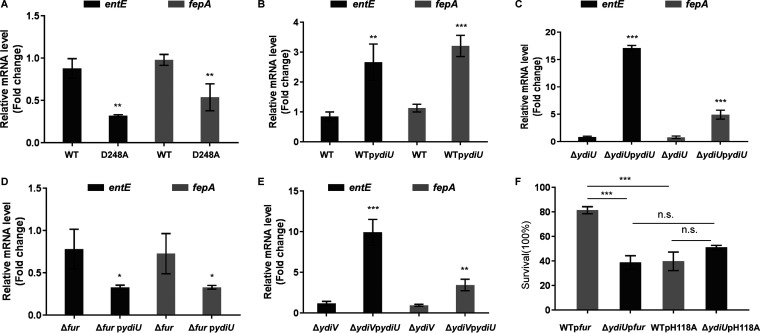
Regulation of iron uptake by YdiU is dependent on the UMPylation activity and Fur. (A to E) The transcription levels of iron uptake genes (*entE* and *fepA*) in Salmonella strains cultured under iron deficiency conditions were detected by qRT-PCR. (A) The mRNA level of *entE* and *fepA* in WT and YdiU D248A mutant strains. (B) The mRNA level of *entE* and *fepA* in WT and WT pYdiU strains. (C) The mRNA level of *entE* and *fepA* in Δ*ydiU* and Δ*ydiU* p*ydiU* strains. (D) The mRNA level of *entE* and *fepA* in ΔFur and Δ*fur* p*ydiU* strains. (E) The mRNA level of *entE* and *fepA* in Δ*ydiV* and Δ*ydiV* p*ydiU* strains. (F) The survival rates of WT p*fur*, Δ*ydiU* p*fur*, WT p*fur*H118A, and Δ*ydiU* p*fur*H118A strains after metal deprivation. The above-described experiments were performed as three replicates, and the mean values are presented. ***, *P* < 0.001; **, *P* < 0.01; *, *P* < 0.05; n.s., *P* > 0.05.

A YdiU D248A mutant Salmonella strain was constructed as a strain in which YdiU lacks UMPylation activity (28). Comparing the wild-type and D248A mutant strains, we found that the expression of iron uptake genes in mutant bacteria was significantly decreased compared to that in wild-type bacteria under iron-deficient conditions, indicating that the regulation of iron uptake by YdiU was dependent on its UMPylation activity (Fig. 5A). To further explore the effect of YdiU on iron uptake, a vector allowing strong and constitutive expression of the *ydiU* gene was constructed and transformed into WT, Δ*ydiU*, and Δ*fur* strains (WT p*ydiU*, Δ*ydiU* p*ydiU*, and Δ*fur* p*ydiU* strains, respectively). The expression levels of *entE* and *fepA* were determined by qPCR for the original strains and YdiU-expressing strains under the iron-deficient condition (Fig. 5B to D). The plasmid-driven expression of YdiU in the WT and Δ*ydiU* strains dramatically increased the expression of iron uptake genes; however, in contrast, the expression of YdiU slightly decreased the expression of iron uptake genes in the Δ*fur* strain, suggesting that activation of iron uptake by YdiU depended on Fur. Our previous study showed that *ydiV*, the gene next to *ydiU* on the chromosome, modulates iron homeostasis by changing the conformation of Fur with the assistance of SlyD in Escherichia coli (27). To test the potential role of YdiV in YdiU-mediated iron regulation in Salmonella, we constructed Δ*ydiV* and YdiU-expressing Δ*ydiV* (Δ*ydiV* p*ydiU*) strains and detected the mRNA levels of *entE* and *fepA* (Fig. 5E). The results showed drastically activated expression levels of *entE* and *fepA* by YdiU in both strains, suggesting that regulation of iron uptake by YdiU was independent of YdiV. To further determine the impact of YdiU on Fur-mediated inhibition of iron uptake, we constructed Fur-expressing WT and Δ*ydiU* strains, WT p*fur* and Δ*ydiU* p*fur*, respectively. We challenged WT p*fur* and Δ*ydiU* p*fur* strains with iron deficiency stress and observed significantly decreased survival for Δ*ydiU* p*fur* compared with that of WT p*fur* (Fig. 5F). To determine the role of H118-specific UMPylation, WT and Δ*ydiU* Fur H118A-overexpressing strains were constructed and treated with iron deficiency stress (Fig. 5F). The survival rate of WT p*fur*H118A displayed a significant reduction compared to WT p*fur*, with no statistical difference observed between WT p*fur*H118A and Δ*ydiU* p*fur*H118A. Collectively, these results suggest that activation of iron uptake by YdiU was indeed achieved by blocking the inhibitory effect of Fur on iron uptake through UMPylation of Fur on H118.

### YdiU-mediated regulation of iron uptake facilitates Salmonella infection.

**FIG 6:**
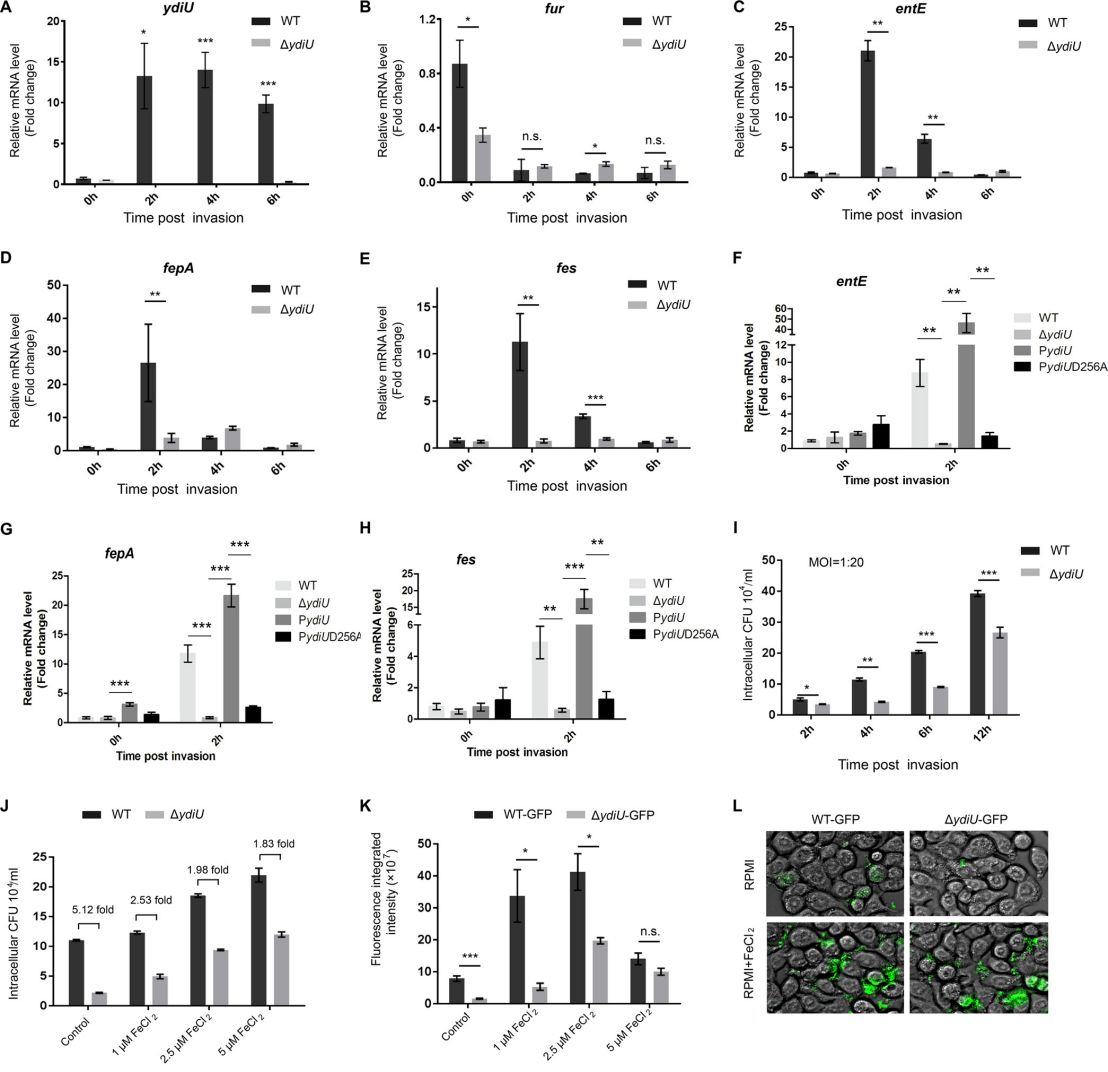
YdiU effectively activates Salmonella iron uptake within host cells. (A) Salmonella upregulates YdiU expression during invasion. The mRNA levels of *ydiU* before and after Salmonella invasion were detected in WT and Δ*ydiU* strains by qRT-PCR. (B) YdiU did not inhibit the expression of Fur. The mRNA levels of fur before and after Salmonella invasion were detected in WT and Δ*ydiU* strains by qRT-PCR. (C to E) The mRNA levels of *entE* (C), *fepA* (D), and *fes* (E) before and after Salmonella invasion were detected in WT and Δ*ydiU* strains by qRT-PCR. The transcription levels of *entE* (F), *fepA* (G), and *fes* (H) before and after WT, Δ*ydiU*, Δ*ydiU* p*ydiU*, and Δ*ydiU* p*ydiUD256A* entering HT29 cells were detected by qRT-PCR. (I) The intracellular amounts of bacteria were quantified in HT-29 cells at the indicated times postinfection. (J) The intracellular amounts of WT and Δ*ydiU* strains were quantified in HT-29 cells cultured with different concentrations of FeCl_2_. (K) The fluorescence integrated intensity was applied and quantified with GFP-expressing Salmonella-infected HT-29 cells cultured in medium with different concentrations of FeCl_2_. (L) HT-29 cells infected with GFP-expressing WT and Δ*ydiU* strain cultured in the medium with or without FeCl_2_. Experiments were performed with at least three replicates, and the mean values are presented. ***, *P* < 0.001; **, *P* < 0.01; *, *P* < 0.05; n.s., *P* > 0.05.

To investigate the function of YdiU during host infection of Salmonella, HT-29 cells were infected with WT or Δ*ydiU* strains at the same multiplicity of infection (MOI). HT29 cells were lysed 2 h, 4 h, and 6 h postinfection, and then the expression levels of Salmonella *ydiU*, *fur*, and iron uptake genes were detected by qPCR (Fig. 6). Compared with the sample prior to invasion, the expression of *ydiU* increased 13.26-, 14.01-, and 9.85-fold 2, 4, and 6 h after WT Salmonella invasion, with no expression of *ydiU* detected in the Δ*ydiU* strain (Fig. 6A). The expression of *fur* in the Δ*ydiU* strain did not significantly differ from that of the WT strain during infection (Fig. 6B), while the expression of iron uptake genes in Δ*ydiU* strain was significantly decreased compared with the WT strain (Fig. 6C to E). Consistent with previous studies (36), the expression of iron uptake genes in WT strain increased 10- to 20-fold when measured 2 h postinvasion (21.03-fold for *entE*, 26.50-fold for *fepA*, and 11.27-fold for *fes*). However, the expression of iron uptake genes did not significantly increase in the Δ*ydiU* strain after invasion (1.63-fold for *entE*, 3.8-fold for *fepA*, and 0.74-fold for *fes*), demonstrating that YdiU is required for efficient activation of iron acquisition during Salmonella infection. As further confirmation, we conducted complementation experiments of Δ*ydiU* strain with native *ydiU* or the *ydiU* D256A mutant. As expected, the insufficient expression levels of iron uptake genes (*entE*, *fepA*, and *fes*) in Δ*ydiU* mutant within host cells can be rescued by native YdiU but not by the YdiU D256A mutant (Fig. 6F to H).

Given that acquisition of iron is a prerequisite for Salmonella survival in host cells (21, 34), we hypothesize that YdiU facilitates Salmonella survival within host cells. To test this hypothesis, we measured the survival of WT and Δ*ydiU* strains at various times after entry of Salmonella into HT-29 cells. The amounts of intracellular bacteria in host cells were determined by counting CFU 2 h, 4 h, 6 h, and 12 h postinvasion. Compared with the WT strain, the Δ*ydiU* strain showed significantly attenuated ability to colonize in HT-29 cells (Fig. 6I). When iron was added to the cell culture, the survival difference between WT and Δ*ydiU* strains decreased from 5.12-fold to 1.83-fold, suggesting that the decline in survival of Δ*ydiU* strain within host cells was partially caused by the deficiency in iron uptake (Fig. 6J). To further investigate the effect of YdiU on Salmonella infection *in vivo*, green fluorescent protein (GFP)-expressing WT (WT-GFP) and GFP-expressing Δ*ydiU* (Δ*ydiU*-GFP) strains were constructed and used to infect HT-29 cells. FeCl_2_ was added to the cell culture to observe the effect of iron on intracellular survival of *Salmonella*. Fluorescence intensity was measured to determine bacterial count. The results clearly showed that the addition of FeCl_2_ to cell culture improved survival for both WT-GFP and Δ*ydiU*-GFP strains (Fig. 6K and L). Consistent with Fig. 6J, the imaging results also support the effect of iron addition to improve the inhibited growth of Δ*ydiU*-GFP strain compared with WT-GFP (Fig. 6L).

### H118 of Fur is essential for the activation of iron uptake pathway during Salmonella infection.

**FIG 7:**
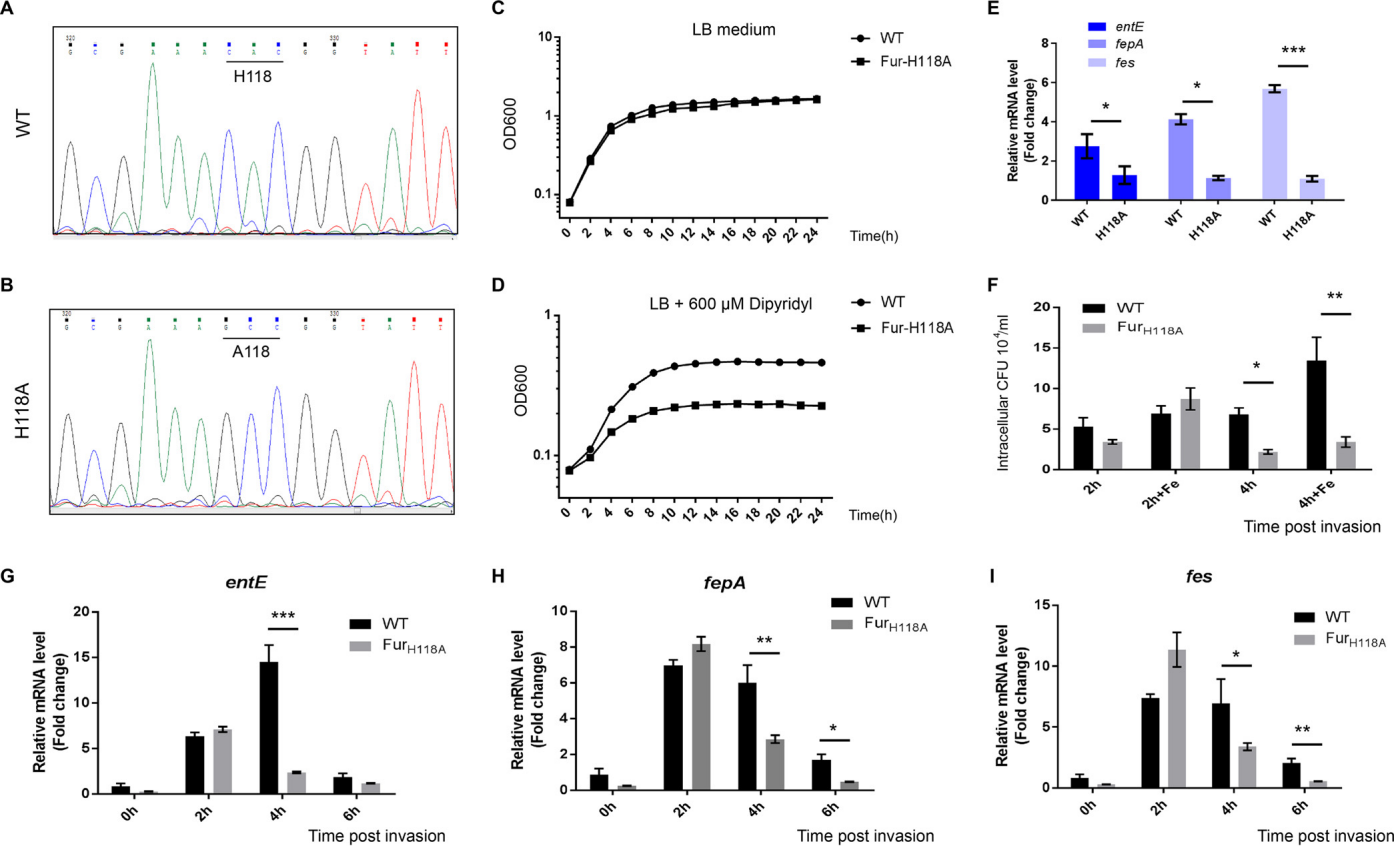
Fur H118 is essential for Salmonella survival under iron deficiency conditions and during infection. (A and B) Sanger sequencing results of wild-type strain (A) and Fur H118A (B) mutant strain. (C and D) The growth curves of WT and H118A mutant strains cultivated in LB medium or metal-limited medium. (E) The transcription levels of iron uptake genes (*entE*, *fepA*, and *fes*) in WT and H118A mutant strains cultured under metal deficiency condition. (F) The intracellular amounts of bacteria were quantified in HT-29 cells at the indicated times postinfection. HT-29 cells were cultured in the medium with or without FeCl_2_. (G to I) The mRNA levels of *entE* (G), *fepA* (H), and *fes* (I) before and after invasion were detected in WT and H118A mutant strains. Experiments were performed in at least three replicates, and the mean values are presented. ***, *P* < 0.001; **, *P* < 0.01; *, *P* < 0.05; n.s., *P* > 0.05.

To further investigate the function of Fur UMPylation on iron metabolism, a single nucleotide polymorphism was inserted into wild-type Salmonella to generate a Fur H118A mutant strain that cannot be regulated by YdiU-mediated UMPylation, so it would restrict Fur in the deUMPylated state (Fig. 7A and B). Similar to what we observed in the Δ*ydiU* strain (Fig. 1F and G), a serious growth defect of Fur H118A was observed under metal-deficient conditions but not in LB medium (Fig. 7C and D). The expression levels of iron uptake genes in WT and Fur H118A strain under iron deficiency conditions were detected by qPCR (Fig. 7E). Compared with the WT strain, there was dramatically decreased transcription of iron uptake genes in the H118A strain under iron-deficient conditions: 0.36-fold for *entE*, 0.24-fold for *fepA*, and 0.18-fold for *fes*. The numbers of CFU of WT and H118A strains were determined 2 h and 4 h postinvasion into HT-29 cells (Fig. 7F). No significant difference was observed 2 h postinvasion; however, the H118A strain exhibited significantly attenuated survival in HT-29 cells 4 h postinvasion compared with the WT strain (Fig. 7F). Additionally, the expression of iron uptake genes in the H118A strain was markedly reduced compared with the WT strain 4 h postinfection: 0.16-fold for *entE*, 0.48-fold for *fepA*, and 0.50-fold for *fes* (Fig. 7G to I). The above-described data indicate that restricting Fur in the deUMPylated state dramatically eliminates Salmonella iron uptake under iron deficiency environments and therefore limits Salmonella proliferation within host cells.

## DISCUSSION

When bacteria enter host cells, obtaining enough iron from the host is critical for bacterial colonization and intracellular survival (7, 20). The Fur protein is the most critical negative regulator of bacterial iron adsorption (17, 39, 41). Interestingly, when bacteria enter host cells, the expression of Fur remains high, but bacteria can upregulate the expression of iron absorption-related genes 10- to 20-fold to rapidly initiate iron absorption (36, 37). According to the classical model, iron ions regulate Fur binding to promoters of iron absorption genes. When the iron ion concentration is low, Fur protein is released from its target DNA sequences, allowing RNA polymerase to bind and transcribe the iron absorption genes (18). However, there are limitations of this simple iron regulation model. First, both *in vivo* and *in vitro* studies show that Fur can bind to iron, manganese, and nickel ions, and all three allow DNA binding activity (25). Bacteria can absorb large amounts of extracellular manganese ions when they enter an iron-deficient condition, increasing the intracellular manganese ion concentration up to 20-fold (42, 43). Therefore, it may not be possible to completely regulate Fur protein function only by control of iron concentration. YdiU-mediated UMPylation of Fur probably works in this event. YdiU is an obligatory Mn-dependent UMPylator. The intracellular Mn^2+^ concentration of bacteria is actually elevated in an iron-deficient environment, and UMPylation of Fur by YdiU is initiated by Mn^2+^. Since Fur can bind Mn and Fe ions indistinguishably, Mn can metallate Fur and activate DNA binding by Fur. UMPylation might function as a regulatory switch to effectively prevent Mn-activated iron uptake inhibition of Fur. YdiU may be a key link allowing Salmonella to sense the Mn/Fe ratio and turn on the low-Fe regulatory response.

Previous studies have found that the EAL-like protein YdiV can transform Fur into a state that cannot bind to DNA with the help of SlyD (27). YdiV is next to YdiU on the chromosome, and here we found that YdiU can inhibit Fur's DNA binding activity by posttranslational modification, thus rapidly promoting iron absorption. YdiV and YdiU have similar functions, as both proteins turn off flagellar synthesis and initiate iron uptake. Interestingly, YdiV and YdiU exert their functions through completely different mechansims, YdiV through protein interaction and YdiU through UMPylation, suggesting these two proteins function independently of each other. However, since these two genes are close to each other on the chromosome, they may be regulated by the same transcription factors (nutrient deficiency induces expression of both YdiU and YdiV), and there will likely be some synergy in expression regulation.

**FIG 8:**
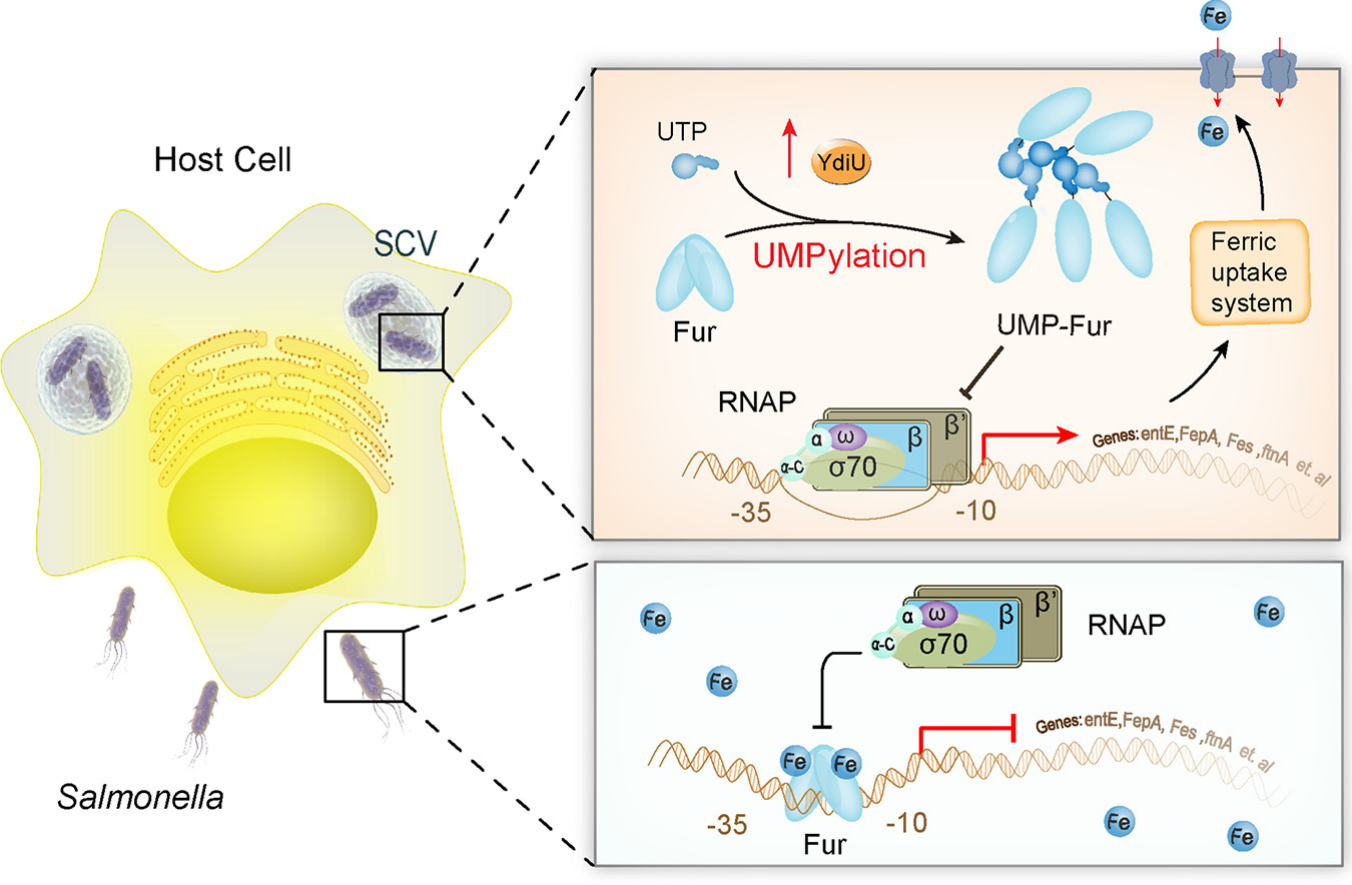
Model of YdiU-mediated iron uptake regulation during Salmonella infection. Before Salmonella enters host cells, iron-bound Fur can inhibit the transcription of iron uptake genes. When Salmonella enters host cells and is subjected to iron-deficient conditions, the expression of YdiU is induced by stress signaling. YdiU then modifies Fur with UMP on its H118 residue. UMPylated Fur is removed from the promoters of iron uptake genes, relieving the inhibition of the iron uptake genes by Fur. Bacteria will take up more iron through iron carriers, increasing survival in host cells.

Based on the results of this study, we propose the following mechanistic model (Fig. 8). When Salmonella grow in an iron-rich environment, the Fur protein binds to the promoter region of the iron absorption gene, inhibiting the recruitment of RNA polymerase and minimizing iron absorption. When Salmonella enter host cells, YdiU protein expression is activated by iron deficiency, high reactive oxygen species, and low pH. YdiU uses UTP to transfer a UMP group to H118 of Fur, preventing Fur dimerization. Fur is displaced from the promoter region of iron absorption genes, thus allowing its activation. Through this mechanism, Salmonella obtain enough iron to survive inside host cells. The identified modification sites are highly conserved in homologous proteins (see Fig. S6 in the supplemental material), suggesting that this Fur regulatory mechanism is widespread. The covalent attachment mode of UMP to histidine is similar to that of phosphorylated histidine (pHis), which plays a crucial role in prokaryotic signal transduction (44, 45). Because pHis is a labile posttranslational modification under acid conditions, a similar acid lability of UMP-His was assumed. Furthermore, we speculate that a de-UMPylase, analogous to pHis phosphatases (46), regulates UMPylated proteins dynamically.

Quantitative proteomic analysis demonstrated YdiU regulated cell motility, iron homeostasis, virulence, and energy production in Salmonella under iron deficiency conditions (Fig. S1). Previous studies showed that Fur is not only a key regulator of iron absorption but also indirectly affects expression of 20% of bacterial proteins and is involved in pathogenicity, acid tolerance, and nitrate respiration of Salmonella (47–49). Thus, it is possible that regulatory activities of YdiU in addition to iron homeostasis are controlled by the UMPylation of Fur. To our knowledge, UMPylation is the first posttranslational modification found to regulate Fur function. Fur was found to be UMPylated at H33 in our previous study, and H118 was not identified as a site of UMPylation in those data (28). The UMPylated peptides in our 2020 study were identified from proteomics data using whole bacterial proteins, and this may explain the lack of detection of Fur H118 UMPylation. In this study, UMPylation of Fur H118 was observed both *in vitro* and *in vivo*, and more importantly, the single mutation of H118 led to a significant decrease in bacterial survival under iron-deficient conditions, demonstrating H118 is a critical site for Fur activity. According to these observations, we propose that posttranslational modification plays a crucial role in the regulation of Fur.

## MATERIALS AND METHODS

### Bacterial strains and culture conditions.

All bacterial strains are listed in Table S1 in the supplemental material. Generally, strains were propagated in Luria-Bertani medium at 37°C with shaking at 200 rpm, and LB agar plates contained 1.5% (wt/vol) agar supplemented with antibiotics and 2,2′-dipyridyl as required. For iron stress experiments, the strains were activated in LB medium overnight, and then cultures were transferred to fresh medium at an OD_600_ of 0.01 for homogenized growth. When strains entered mid-log phase (OD_600 _= 0.6), 2,2′-dipyridyl was added at the indicated concentrations. For the experiments shown in Fig. 1A and B and 2E, log-phase Salmonella cultures were treated with different concentrations of 2,2′-dipyridyl (200 to 600 μM) for 6 h. For the experiments shown in Fig. 1C and D and 2F, when strains entered mid-log phase, 600 μM 2,2′-dipyridyl was added to induce gene expression, and samples with no 2,2′-dipyridyl were used as a control (0 h). The cultures were sampled at 1 h, 2 h, or 3 h. For the experiments shown in Fig. S2 and S3C and Fig. 5A to E and 7E, log-phase Salmonella cultures were treated with 600 μM 2,2′-dipyridyl for 3 h.

10.1128/mbio.00207-22.8 TABLE S1 Strains used in this study. Download Table S1, DOCX file, 0.02 MB. Copyright © 2022 Jia et al. 2022 Jia et al. https://creativecommons.org/licenses/by/4.0/ This content is distributed under the terms of the Creative Commons Attribution 4.0 International license.

### RNA extraction and qRT-PCR.

Total RNA was isolated using SPARKeasy Bacteria RNA kit (Sparkjade) or RNAprep Pure Cell/Bacteria kit (Tiangen). Reverse transcription reactions were performed using the RevertAid cDNA synthesis kit (Thermo) according to the manufacturer’s instructions. Quantitative real-time-PCR (qRT-PCR) reactions were performed on an Applied Biosystems 7500 sequence detection system (Applied Biosystems) using iTaq Universal SYBR green supermix (Bio-Rad).

### Western blotting.

Bacterial cells were lysed using 1× loading buffer and then heated at 95°C for 10 min before SDS-PAGE. Total proteins were examined by 12% SDS-PAGE and then electrotransferred onto a polyvinylidene fluoride (PVDF) membrane (Millipore). The membranes were blocked with 5% milk in phosphate-buffered saline plus Tween (PBST) at 25°C for 1 h, followed by incubation overnight at 4°C with polyclonal antibody to YdiU (1:2,000 dilution) or GapA (1:10,000 dilution) in PBST. After three washes with PBST, the membranes were then incubated at 25°C for 1 h with horseradish peroxidase (HRP)-conjugated goat anti-rabbit or mouse IgG (Abcam) diluted 1:10,000 in PBST. Finally, the membranes were incubated with a chemiluminescent substrate (Immobilon Western HRP substrate; Millipore) and detected using a FluorChem imager (Uvitec).

### Survival rate under metal deficiency condition.

Wild-type and Δ*ydiU* strains were grown to logarithmic phase in LB medium, and then 2,2′-dipyridyl was added to the bacterial solution to a final concentration of 800 μM. Samples were removed after exposure to 2,2′-dipyridyl for 3 h and then serially diluted into PBS (pH 7.4). For the experiments shown in Fig. 1E and 5F, 250-μL samples of 10^6^ bacteria were plated on LB agar plates. Plates were incubated at 37°C for 24 h and the colonies were counted. The percentage of bacterial survival was determined (with time zero representing 100%) as a function of the duration of iron deficiency.

### Growth under metal-deficient condition.

For the experiments shown in Fig. 1F and G and 7C and D, bacterial growth was measured using a Bioscreen C automatic growth analyzer at 37°C with a honeycomb microplate. To measure growth, bacteria were diluted into LB medium plus 400 μM 2,2′-dipyridyl, 300 μL of diluted culture were added into individual wells, and each strain was assayed in triplicate. Samples were measured at 600-nm absorbance every 5 min for 24 h. For the iron-add-back experiments shown in Fig. 1H, WT and Δ*ydiU* Salmonella cells were cultured in LB medium plus 600 μM 2,2′-dipyridyl. Two hours after the start of culturing, different concentrations of FeCl_2_ were added back to the medium, and the OD_600_ values were monitored in real time.

### Sample preparation for mass spectrometry-based proteomic analysis.

Wild-type and Δ*ydiU* strains were grown to logarithmic phase in LB medium, and then 2, 2′-dipyridyl was added to the bacterial solution to a final concentration of 600 μM and allowed to culture at 37°C for 3 h. The bacteria were then pelleted by centrifugation and lysed using a buffer containing 8 M urea, 1% Triton X-100, 10 mM dithiothreitol, and 2 mM EDTA. After centrifugation of the samples at 20,000 × *g* for 10 min, the supernatants were removed and the amount of protein quantified with the bicinchoninic acid (BCA) protein assay kit (Beyotime). Samples of 200 μg protein were purified by ultrafiltration, incubated with iodoacetamide to block reduced cysteine residues, and then digested with 4 μg trypsin at 37°C overnight. Peptides of each sample were desalted on C_18_ cartridges, concentrated by vacuum centrifugation, and reconstituted in 40 μL of 0.1% (vol/vol) formic acid.

### TMT quantitative proteomic analysis.

Tandem mass tag (TMT) quantitative proteomic analysis was performed by Jingjie Biological Technology Co., Ltd. (Hangzhou, China). In brief, total protein was extracted from wild-type and Δ*ydiU* cells using a high-intensity ultrasonic processor (Scientz) in lysis buffer (8 M urea, 1% protease inhibitor cocktail; Roche). The cell lysates were collected and quantified with the BCA protein assay kit (Beyotime) according to the manufacturer’s instructions. For trypsin digestion, the protein solution was reduced with 5 mM dithiothreitol and then alkylated with 11 mM iodoacetamide according to a previous study (50). The digested samples then were desalted by a Strata X-C18-SPE column (Phenomenex, Torrance, CA, USA) and vacuum dried. For TMT labeling, we reconstituted peptides in 0.5 M TEAB (TMT kit; Thermo Scientific, Waltham, MA, USA) and processed them according to the manufacturer’s protocol. For sample preparation, the tryptic peptides were fractionated by high-pH (using a gradient of 8% to 32% acetonitrile, pH 9.0), reverse-phase high-performance liquid chromatography (HPLC) using an Agilent 300Extend C_18_ column (5-μm particles, 4.6-mm inner diameter, 250-mm length). Running of samples in liquid chromatography-tandem mass spectrometry (LC-MS/MS) and analysis were performed as described before (51).

### ICP-MS.

Salmonella strains were inoculated into iron-rich LB medium or iron-limited LB medium (final concentration of 600 μM 2,2′-dipyridyl was added) and cultured to an OD_600_ of 1.0. Cells were collected and washed twice using PBS solution supplemented with 5 mM EDTA to remove ions from the medium and then washed twice using PBS (without EDTA) to remove EDTA. Finally, the cells were dried at 60°C overnight. Next, 0.2-g samples were weighed and transferred to a Teflon digestion tank together with 5 mL nitric acid. After the reaction was complete, the cover was sealed and put into a microwave digestion instrument. After the temperature cooled to below 50°C, the digestion tank was removed, moved to a fume hood, and opened. The sample was resuspended in ultrapure water and transferred to a 50-mL volumetric flask, with three to four cycles of moistening and washing. Samples were then diluted in 20 mL of deionized water and filtered for final analysis using inductively coupled plasma mass spectrometry (ICP-MS) (Thermo iCAPQ). Three biological replicates were performed per sample.

### Generation of constructs and strains.

All plasmids used in this study are listed in Table S2. Salmonella Fur and FurH118A genes were cloned into the pBAD24 vector for *in vivo* study. For biochemical study, *ydiU* and *fur* genes were amplified from *E. coli* K-12 substrain MG1655 genomic DNA and cloned into the pGL01 vector, which is a modified expression vector based on pET15b with a PPase cleavage site to remove the His tag. Mutants of FurH118A were constructed using the Mut Express II fast mutagenesis kit V2 (Vazyme, Nanjing) and separately cloned into pBAD24 and pGL01.

10.1128/mbio.00207-22.9 TABLE S2 Plasmids used in this study. Download Table S2, DOCX file, 0.02 MB. Copyright © 2022 Jia et al. 2022 Jia et al. https://creativecommons.org/licenses/by/4.0/ This content is distributed under the terms of the Creative Commons Attribution 4.0 International license.

The gene knockout mutant of Fur was constructed using the lambda Red recombinase system as described previously (52). YdiU D248A mutant strain and Fur H118A mutant strain were constructed by CRISPR/Case9 with the help of Guangzhou Ubigene Biosciences Co., Ltd.

### Protein expression and purification.

Both YdiU and Fur used for purification were from *E. coli*. Proteins were expressed in E. coli BL21(DE3) or *E. coli* BL21(DE3) *ydiU* knockout strain. Expression and purification of E. coli YdiU were performed as described previously (53). To obtain Fur without UMPylation, the E. coli BL21(DE3) *ydiU* knockout strain was used. Briefly, when the OD_600_ reached 0.6, cultures were cooled to 16°C and protein expression was induced overnight by 0.1 mM isopropyl-β-d-thiogalactopyranoside (IPTG). Harvested cells were lysed by sonication, and proteins were purified by Ni^2+^-nitrilotriacetic acid (NTA) affinity column. The His tag of the proteins was removed by PPase treatment (54), and then proteins were concentrated and purified by Superdex 200 chromatography. To obtain Fur with UMPylation, Fur and YdiU were coexpressed in E. coli BL21(DE3) and purified as described above.

### *In vitro* UMPylation assay with biotin-UTP.

*In vitro* UMPylation assays were performed as described previously (28). Briefly, 4 μg of purified E. coli Fur or FurH118A was incubated with or without 1 μg E. coli YdiU^475^ in a 20-μL reaction buffer containing 25 mM Tris-HCl (pH 7.5), 100 mM NaCl, 10 mM MnCl_2_, and 200 μM biotin-16-UTP at 30°C for 1 h. The Streptavidin HRP blot then was performed as previously described (28).

### Identification of UMPylation sites by MS.

UMPylated proteins were analyzed on 12% NuPAGE gel and stained with Coomassie brilliant blue. The protein bands of E. coli Fur were excised from the gel and digested overnight with trypsin. The digested peptides were desalted using a ZipTip C_18_ column (Millipore, Billerica, MA) for the subsequent LC-MS/MS analysis. About 2 μg of the trypsin-digested peptides was injected into a C_18_ column and eluted with a linear gradient from 5% acetonitrile to 35% acetonitrile, followed by using the Orbitrap Elite mass spectrometer (Thermo Fisher). Data were acquired for the 20 most intense peaks of every full MS scan. MS/MS spectra were searched against the Escherichia coli (strain K-12) database (UniProt no. 83333) using Mascot. MS/MS spectra were searched with a maximum mass tolerance of 10 ppm for the precursors, 0.6 Da for fragments, dynamic UMPylation (306.025302, H_11_C_9_N_2_O_8_P) for Tyr, His, Ser, Thr, and methionine oxidation, and missed cleavage of 2.

### Size-exclusion chromatography.

For the size-exclusion chromatography experiment presented in Fig. 3F, 100 μg E. coli Fur^dYdiU^ and/or 100 μg E. coli Fur^pYdiU^ proteins was analyzed by size-exclusion chromatography using a Superdex 200 column. A 53-kDa protein and a 22-kDa protein were examined by size-exclusion chromatography under the same conditions as controls.

### DLS.

Dynamic light scattering (DLS) was applied to determine the hydrodynamic radius (rH) of protein samples. Measurements were performed in a 96-well plate at 25.0 ± 0.1°C using a DynaPro plate reader 3 (Wyatt Technology, Santa Barbara, California). Proteins were diluted to 0.5 mg/mL, with 100 μL sample per well. Data processing was automatically performed by the software supplied with the instrument.

### Native PAGE.

Proteins were obtained as described above. Native PAGE experiments were carried out by 12% acrylamide gel in native PAGE buffer (pH 8.0; Beyotime Biotechnology) at 120V for 1 h. Modified and unmodified Fur proteins were electrophoretically separated, and the gels were scanned by a calibrated densitometer (GS-900; Bio-Rad) after staining with Coomassie brilliant blue.

### EMSA.

To obtain fluorophore 6-carboxy-fluorescein (FAM)-labeled double-stranded Fur box DNA (GATAATGATAATGATAATGATAATGATAATGA), two reverse complementary single-stranded FAM-Fur box DNA oligonucleotides were mixed to 10 mM in annealing buffer (10 mM Tris-HCl, pH 7.5, 150 mM NaCl), heated at 95°C for 10 min, and slowly cooled to room temperature. Next, 20 nM the annealed DNA was preincubated with different ratios of protein samples at 37°C for 10 min. Samples were resolved on 6% native polyacrylamide gel and electrophoresed in TBE buffer (46 mM Tris base, 46 mM boric acid, 1 mM EDTA, pH 8.0) at 4°C for 60 min at 100 V. Light was avoided in this experiment. Imaging was performed using ChemiDoc MP imaging system (Bio-Rad). Finally, the gel was stained with Coomassie brilliant blue.

### Cell culture and bacterial infection experiments.

The human colon adenocarcinoma cell line HT-29 was maintained at 37°C with 5% CO_2_ in RPMI 1640 medium (Gibco) containing 10% fetal bovine serum (Gibco). Cells were seeded at 1 × 10^7^ in 100-mm tissue culture dishes. Bacteria were cultured in LB medium to OD_600_ of 0.5, diluted into RPMI 1640 medium, and then seeded on HT-29 cells at a multiplicity of infection (MOI) of 20. After 1 h of infection, the bacteria were removed from plates. The cells were washed twice with PBS, and then RPMI 1640 with gentamicin (100 μg/mL) was added to kill the remaining extracellular bacteria. The cells were washed twice, and RPMI 1640 with gentamicin (20 μg/mL) was added to keep the cells alive and inhibit extracellular bacteria. At different time points after infection (2 h, 4 h, or 6 h), cells were washed and lysed in TRIzol reagent (Tiangen) and then stored at −80°C for RNA extraction. Bacteria before invasion were used for the control (0 h). For the experiments shown in Fig. S8, four strains, WT, Δ*ydiU*, Δ*ydiU* p*ydiU*, and Δ*ydiU* p*ydiU*D256A strains, were cultured in LB medium to OD_600_ of 0.2 before addition of l-arabinose to a final concentration of 0.5% to induce YdiU/YdiUD256A expression. After 1 h, cell invasion experiments were performed as described above. For the experiments shown in Fig. 6I and 7F, after infection, cells were washed gently and disrupted with 1% Triton X-100 (Sigma Chemical) at different time points, and then serial dilutions of bacteria were plated on LB agar to determine the number of CFU. For the experiments shown in Fig. 6J, various concentrations of FeCl_2_ (1 μM, 2.5 μM, or 5 μM) were added to RPMI 1640. HT-29 cells cultured without addition of FeCl_2_ were used as a control.

### Fluorescence microcopy.

HT-29 cells were cultured in 96-well format with various concentrations of FeCl_2_. WT and Δ*ydiU* Salmonella expressing GFP were cultured in LB medium to an OD_600_ of 0.5, diluted using RPMI 1640 medium, and seeded on HT-29 cells at an MOI of 20 using previous methods. Fluorescence images were obtained using the Molecular Devices ImageXpress Microconfocal 4 h after infection. After high-throughput image acquisition of the 96-well screening plates (60×; 1 × 10^5^cells per well), automated image analysis for fluorescence intensity was applied by MetaXpress Imaging and Analysis software.

### Statistical analysis.

All experiments were performed with three biological replicates unless otherwise stated. A two-tailed Student's *t* test was used to calculate *P* values using GraphPad Prism or SPSS. Error bars represent the standard errors of the means (SEM). The statistical significance is indicated by *P* values of <0.001 (***), <0.01 (**), or <0.05 (*). Statistical details of experiments are included in the figure legends.

### Data availability.

Proteomic data have been submitted to ProteomeXchange via the PRIDE database (http://www.ebi.ac.uk/pride) under the data set identifier PXD023378.
