# Supplementary figures and images for: Salmonella Facilitates Iron Acquisition through UMPylation of Ferric Uptake Regulator
Source: mBio. 2022 May 9;13(3):e00207-22. doi: 10.1128/mbio.00207-22 (PMC9239237; doi:10.1128/mbio.00207-22)

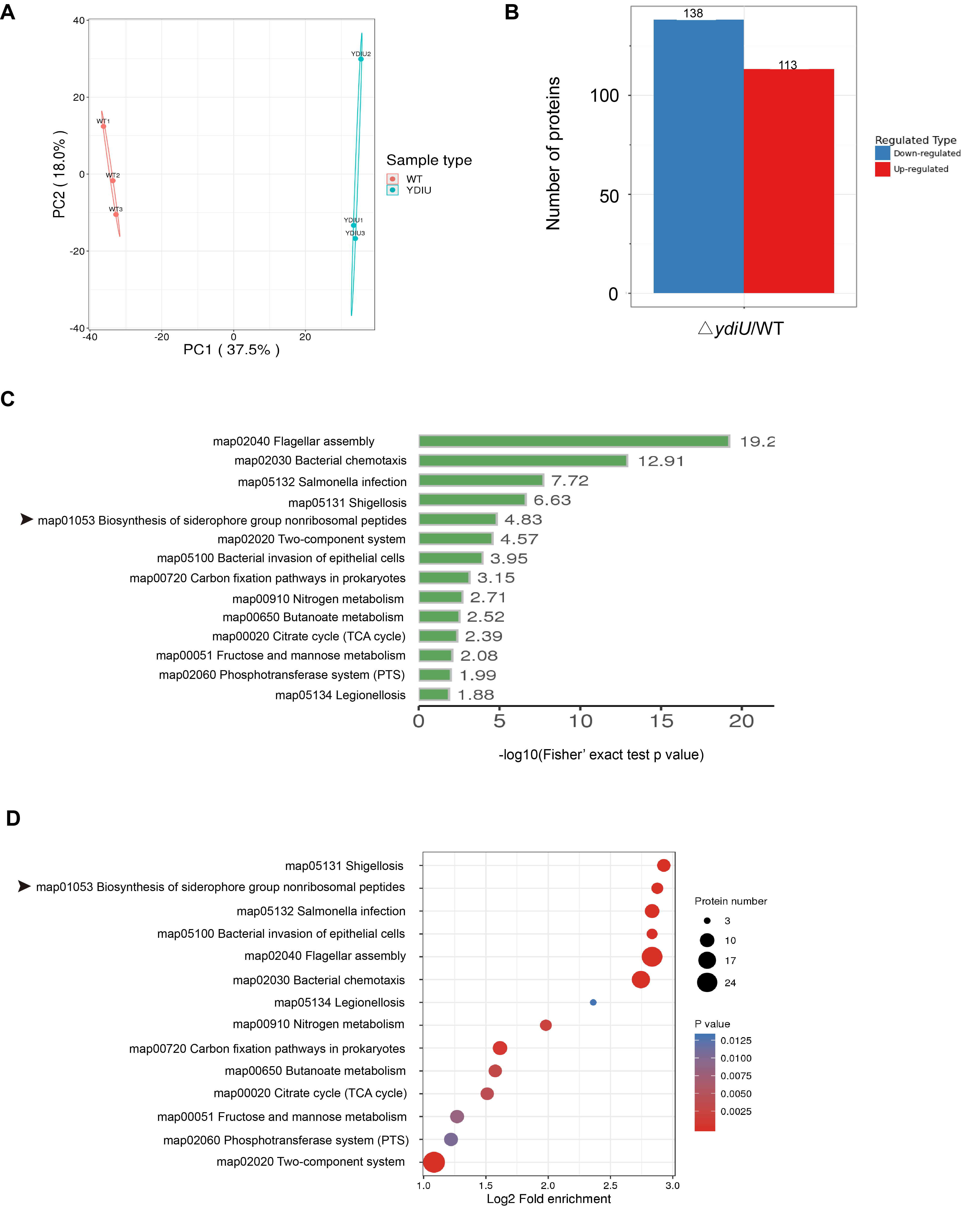

Supplement: FIG S1 [file mbio.00207-22-s0003.tif]

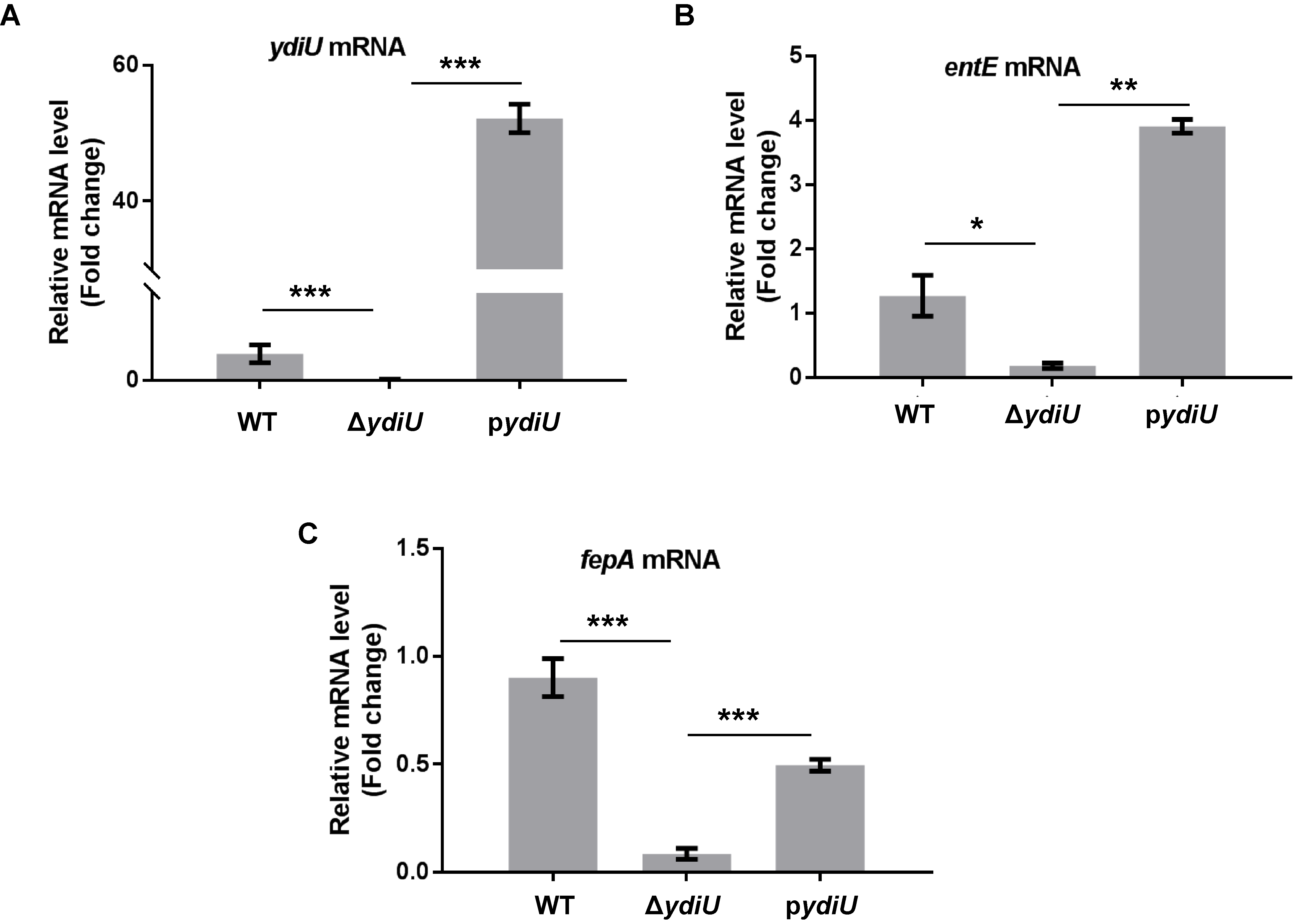

Supplement: FIG S2 [file mbio.00207-22-s0004.tif]

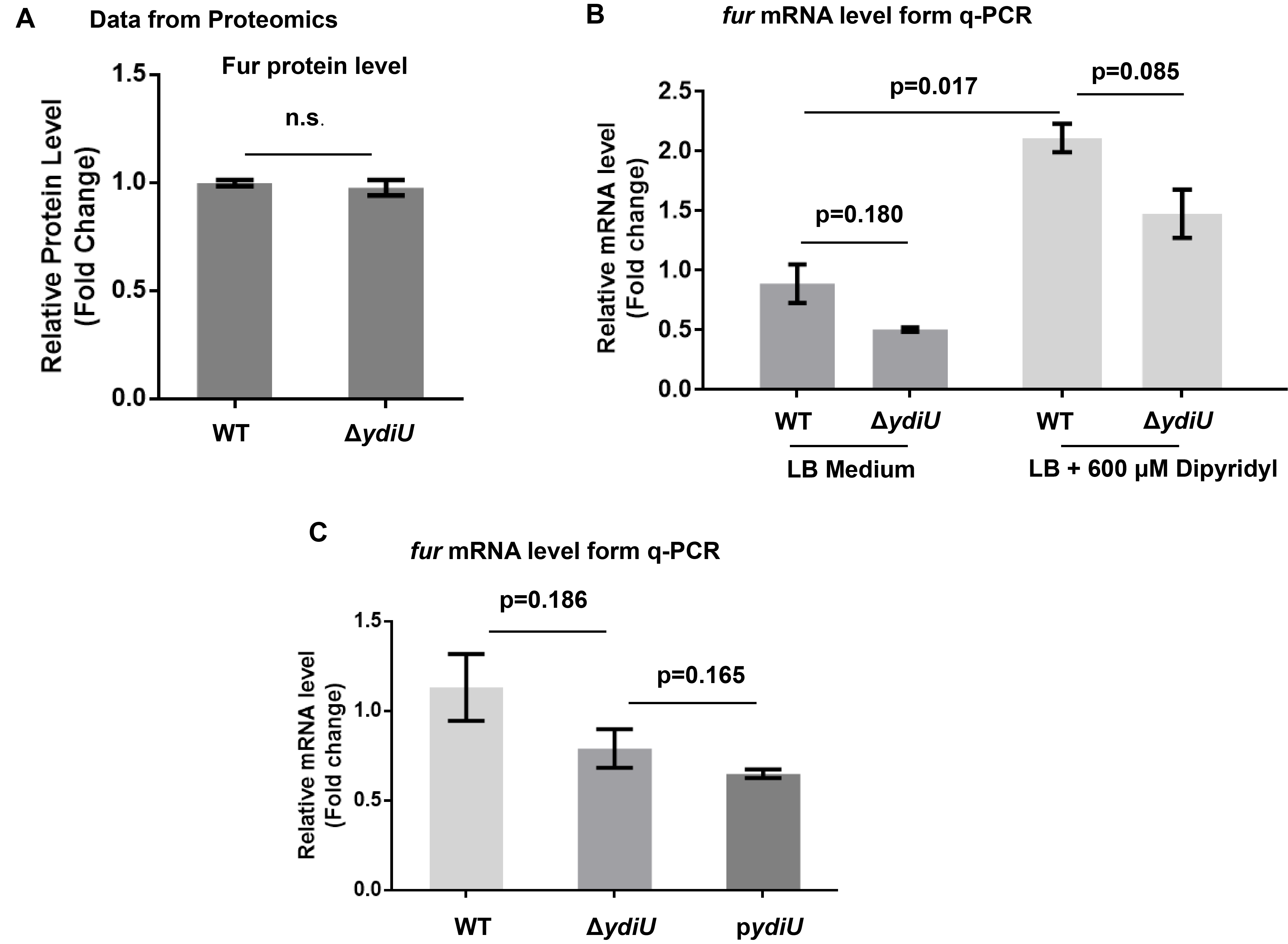

Supplement: FIG S3 [file mbio.00207-22-s0005.tif]

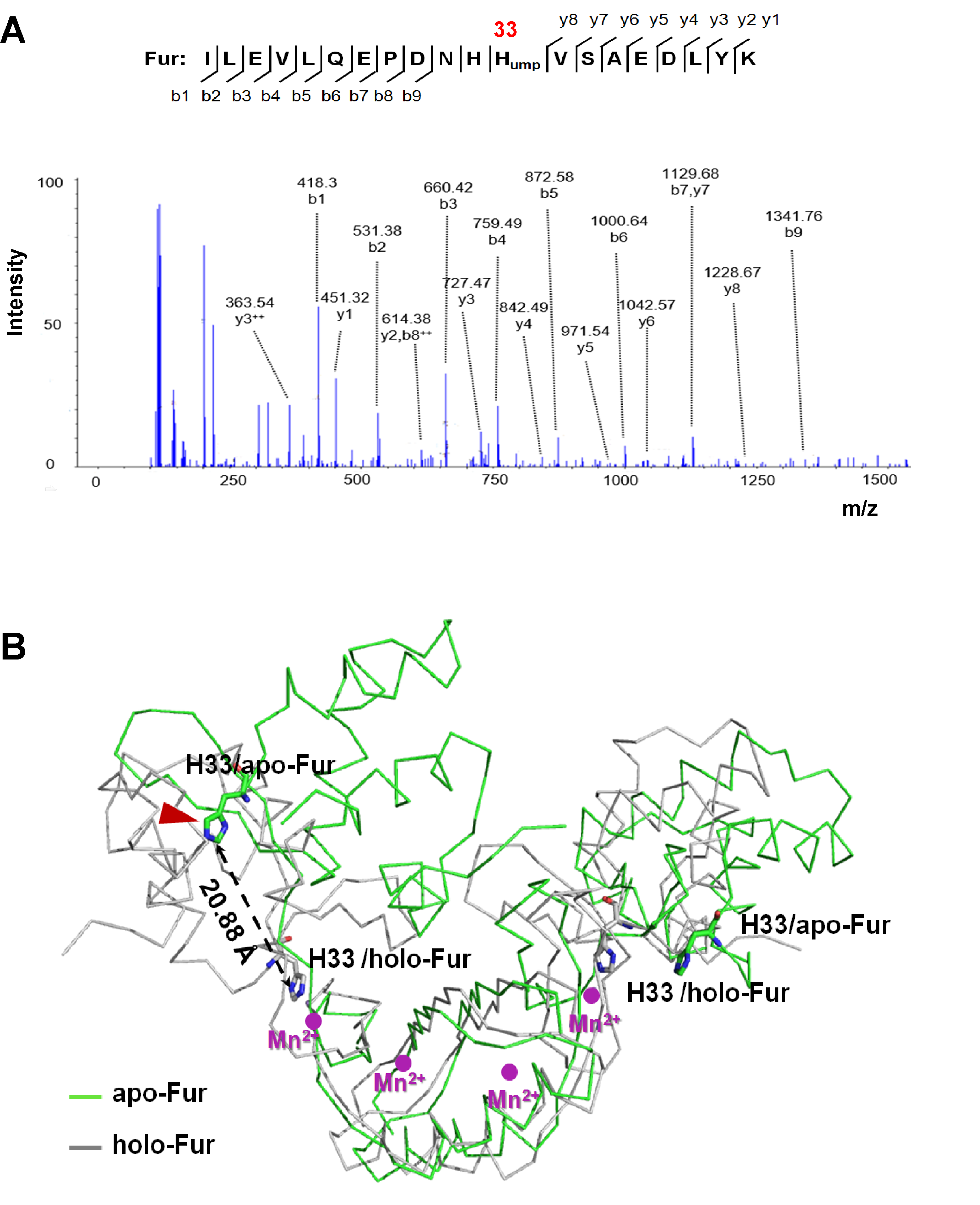

Supplement: FIG S4 [file mbio.00207-22-s0006.tif]

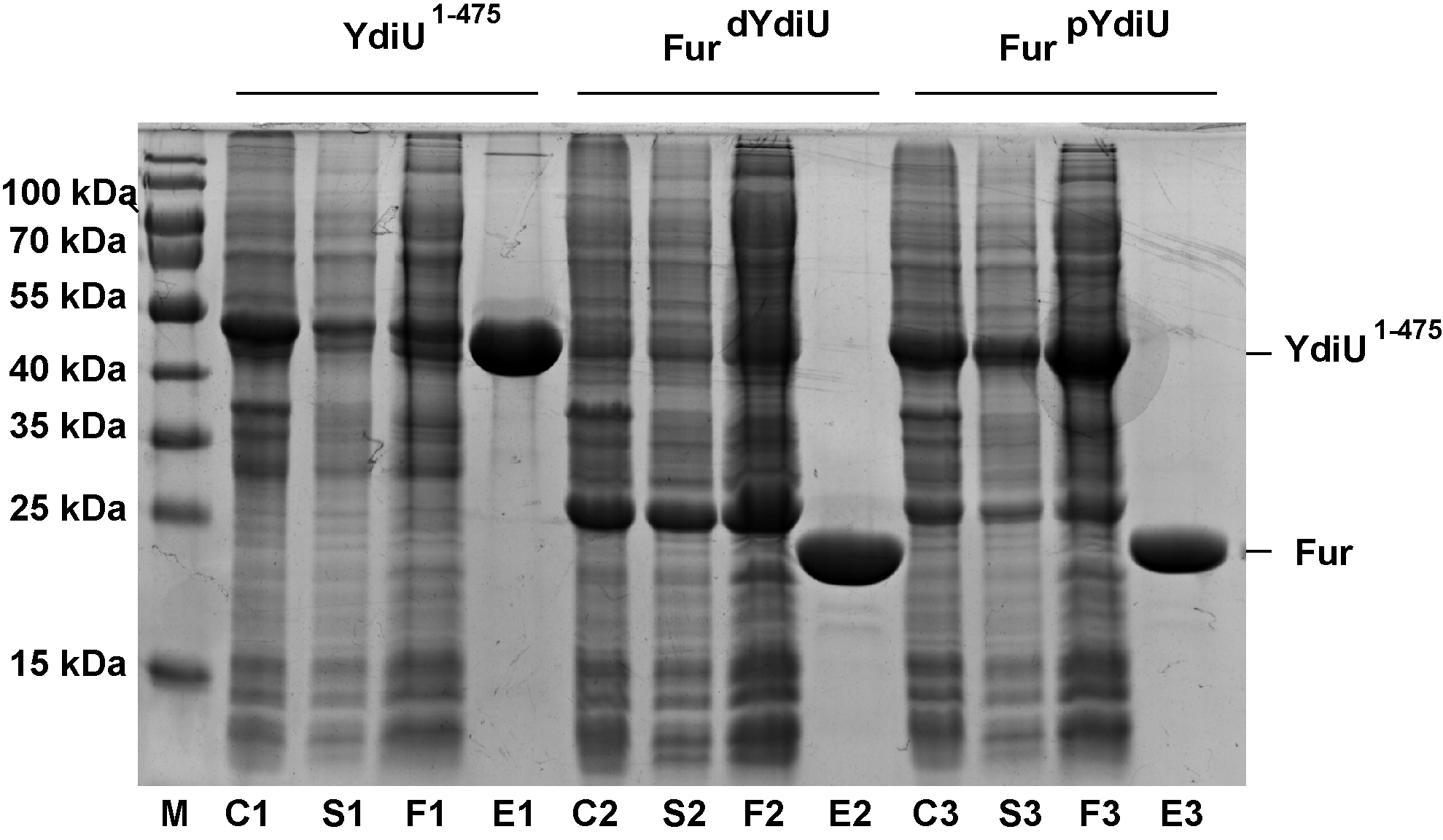

Supplement: FIG S5 [file mbio.00207-22-s0007.tif]

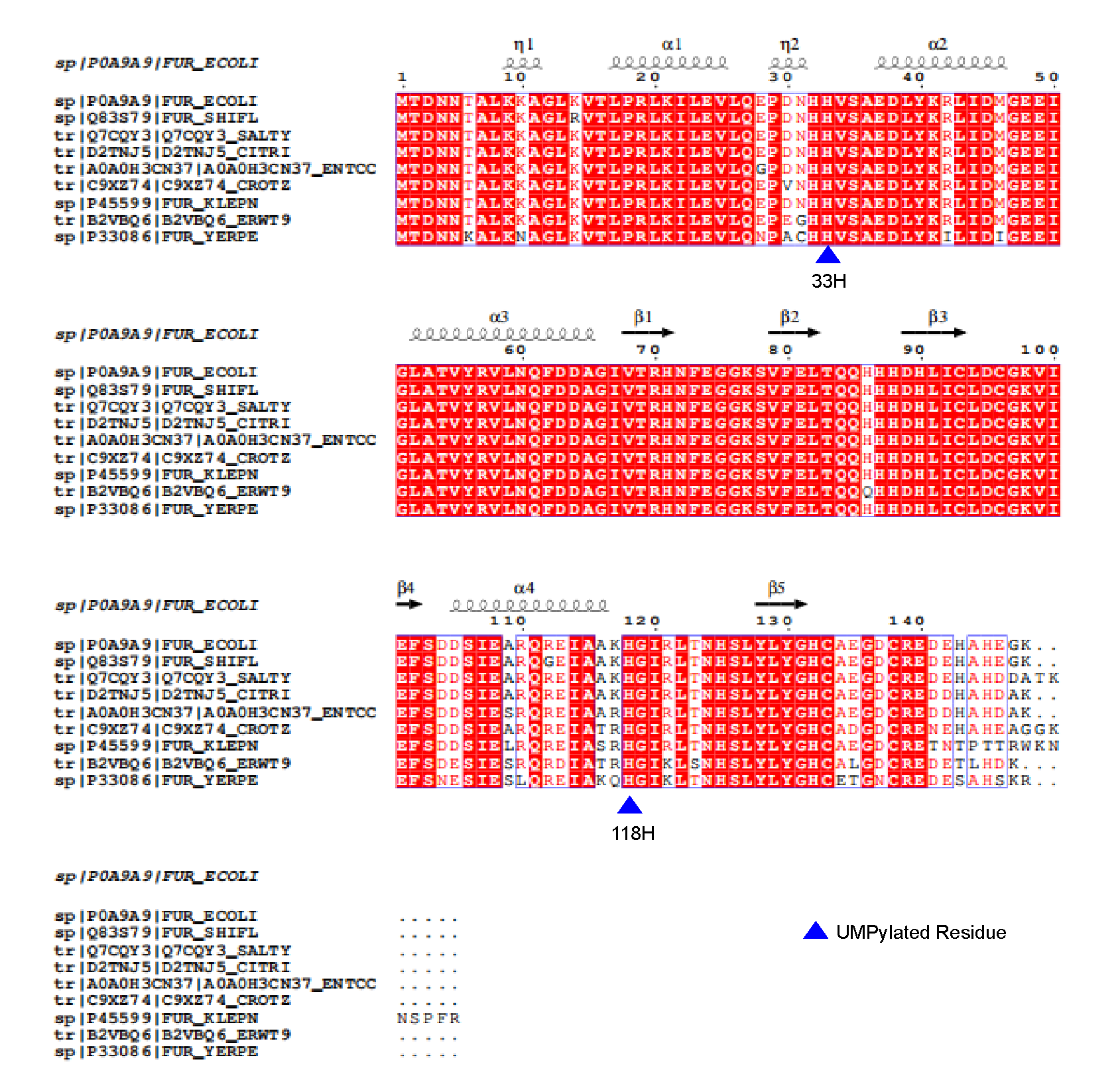

Supplement: FIG S6 [file mbio.00207-22-s0008.tif]

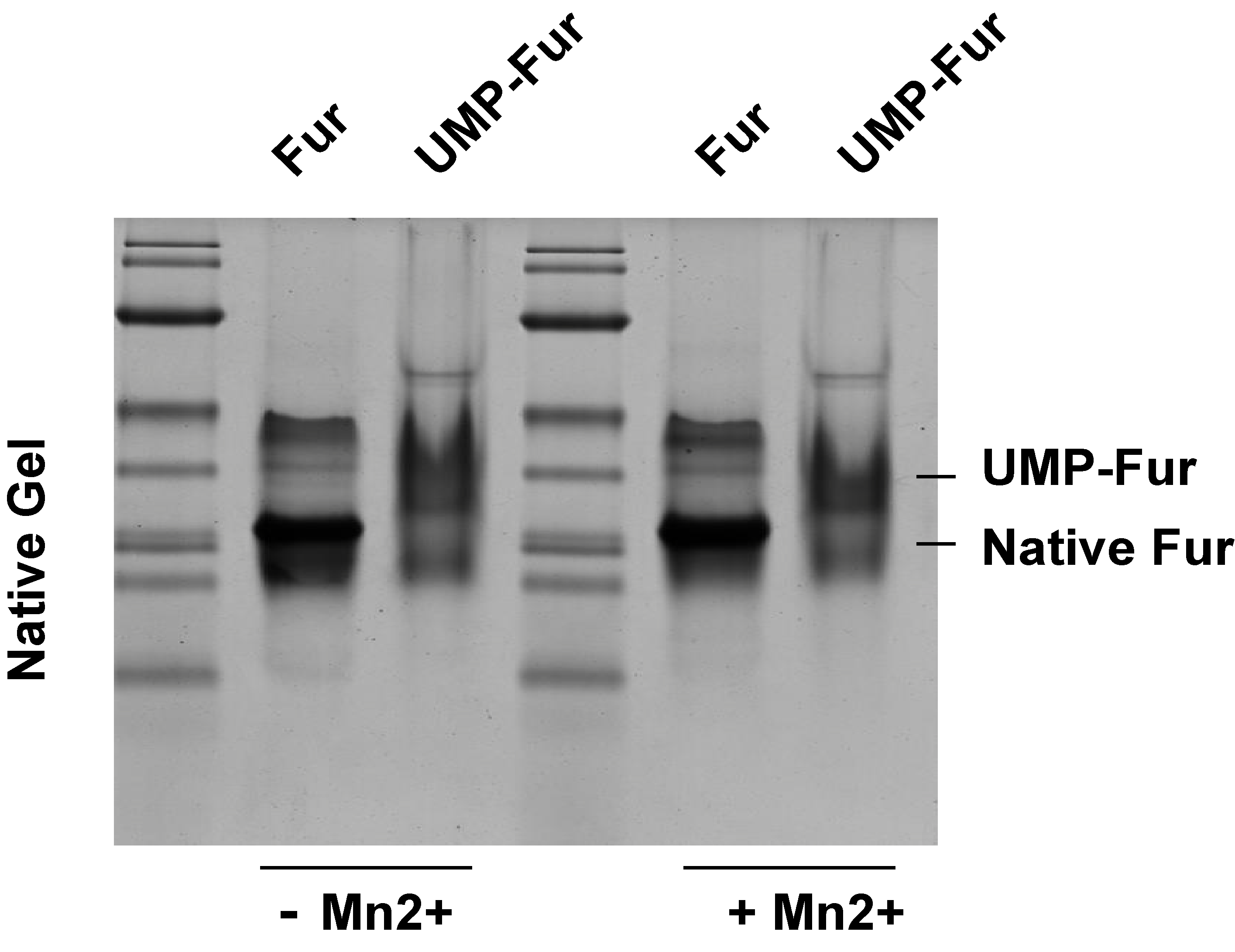

Supplement: FIG S7 [file mbio.00207-22-s0009.tif]
