# Supplementary material for: Salmonella Facilitates Iron Acquisition through UMPylation of Ferric Uptake Regulator
Source: mBio. 2022 May 9;13(3):e00207-22. doi: 10.1128/mbio.00207-22 (PMC9239237; doi:10.1128/mbio.00207-22)
Supplement: TABLE S1 [file mbio.00207-22-s0001.docx]

**Table S1. Strains used in this study**

| No | Strains | Relevant characteristic(s) | Source/Reference |
| --- | --- | --- | --- |
| 1 | WT *Salmonella* | *Salmonella enterica serovar Typhimurium* ATCC14028, no resistance | American Type Culture Collection |
| 2 | WT *Salmonella* VC | Vector pBad24 control, Amp^+^ | [1] |
| 3 | WT *Salmonella* p*ydiU* | YdiU^475^/pBad24, Amp^+^ | [1] |
| 4 | *Salmonella* Δ*ydiU* | *ydiU* knockout strain, no resistance | [1] |
| 5 | *Salmonella* Δ*ydiU* VC | Vector pBad24 control, Amp^+^ | [1] |
| 6 | *Salmonella*Δ*ydiU*p*ydiU* | YdiU^475^/pBad24, Amp^+^ | [1] |
| 7 | *Salmonella*Δ*ydiU*p*ydiU*D256A | YdiU^475^D256A/pBad24, Amp^+^ | [1] |
| 8 | *Salmonella ydiU* D248A | YdiU inactivation strain | This study |
| 9 | *Salmonella* Δ*fur* | *fur* knockout strain, no resistance | This study |
| 10 | *Salmonella* Δ*fur* VC | Vector pBad24 control, Amp^+^ | This study |
| 11 | *Salmonella* Δ*fur* p*ydiU* | YdiU^475^/pBad24, Amp^+^ | This study |
| 12 | *Salmonella* Δ*ydiV* | ydiV knockout strain, no resistance | This study |
| 13 | *Salmonella* Δ*ydiV* VC | Vector pBad24 control, Amp^+^ | This study |
| 14 | *Salmonella* Δ*ydiV*p*ydiU* | YdiU^475^/pBad24, Amp^+^ | This study |
| 15 | WT *Salmonella* p*fur* | Fur/pBad24, Amp^+^ | This study |
| 16 | *Salmonella*Δ*ydiU*p*fur* | Fur/pBad24, Amp^+^ | This study |
| 17 | WT *Salmonella* p*fur* H118A | Fur H118A/pBad24, Amp^+^ | This study |
| 18 | *Salmonella*Δ*ydiU*p*fur* H118A | Fur H118A/pBad24, Amp^+^ | This study |
| 19 | WT *Salmonella* pFPV25.1 | GFP-expressing strain, Amp^+^ | This study |
| 20 | *Salmonella*Δ*ydiU* pFPV25.1 | GFP-expressing strain, Amp^+^ | This study |
| 21 | *E. coli* BL21(DE3) | T7 expression host, no resistance | Takara Bio Inc. |
| 22 | *E. coli* BL21(DE3)Δ*ydiU* | T7 expression host, no resistance | [1] |
| 23 | *E. coli* BL21(DE3)p*ydiU* | YdiU^475^/pGL01, Amp^+^ | [1] |
| 24 | *E. coli* BL21(DE3)Δ*ydiU*p*fur* | Δ*ydiU* Fur/pGL01, Amp^+^ | This study |
| 25 | *E. coli* BL21(DE3) Δ*ydiU*p*fur*H118A | Δ*ydiU* FurH118A/pGL01, Amp^+^ | This study |
| 26 | *E. coli* BL21(DE3)p*fur* p*ydiU* | Fur/pGL01&YdiU^475^/pET29b Amp^+^Kan^+^ | This study |

[1] Yang, Y., Yue, Y., Song, N., Li, C., Yuan, Z., Wang, Y., ... & Li, B. (2020). The YdiU domain modulates bacterial stress signaling through Mn^2+^-dependent UMPylation. *Cell Reports*, 32(12), 108161.
