# Supplementary material for: Salmonella Facilitates Iron Acquisition through UMPylation of Ferric Uptake Regulator
Source: mBio. 2022 May 9;13(3):e00207-22. doi: 10.1128/mbio.00207-22 (PMC9239237; doi:10.1128/mbio.00207-22)
Supplement: TABLE S2 [file mbio.00207-22-s0002.docx]

**Table S2. Plasmids used in this study**

| No | Plasmids | Relevant characteristic(s) | Source/Reference |
| --- | --- | --- | --- |
| 1 | pBad24 | Vector pBad24 control Amp^+^ | [1] |
| 2 | YdiU^475^/pBad24 | *Salmonella* YdiU^1-475aa^ cloned into pBad24 | [2] |
| 3 | YdiU^475^ D256A /pBad24 | *Salmonella* YdiU^1-475aa^ D256A cloned into pBad24 | [2] |
| 4 | Fur/pBad24 | *Salmonella* Fur cloned into pBad24 | This study |
| 5 | Fur H118A/pBad24 | *Salmonella* Fur cloned into pBad24 | This study |
| 6 | pGL01 | Expression Vector Amp^+^ | [3] |
| 7 | YdiU^475^/pGL01 | *E.coli* YdiU 1-475aa cloned into pGL01 | [2] |
| 8 | Fur/pGL01 | *E.coli* Fur 1-148aa cloned into pGL01 | This study |
| 9 | Fur H118A /pGL01 | *E.coli* Fur 1-148aa H118A cloned into pGL01 | This study |
| 10 | pET29b | Expression Vector Kan^+^ | Lab strain |
| 11 | YdiU^475^/pET29b(3T) | *E.coli* YdiU cloned into pET29b | [2] |
| 12 | pFPV25.1 | GFP-expressing Vector Amp^+^ | [4] |

References

[1] Guzman, L. M., Belin, D., Carson, M. J., & Beckwith, J. O. N. (1995). Tight regulation, modulation, and high-level expression by vectors containing the arabinose PBAD promoter. *Journal of bacteriology*, 177(14), 4121-4130.

[2] Yang, Y., Yue, Y., Song, N., Li, C., Yuan, Z., Wang, Y., ... & Li, B. (2020). The YdiU domain modulates bacterial stress signaling through Mn^2+^-dependent UMPylation. *Cell Reports*, 32(12), 108161.

[3] Li, B., Li, N., Wang, F., Guo, L., Huang, Y., Liu, X., ... & Gu, L. (2012). Structural insight of a concentration-dependent mechanism by which YdiV inhibits *Escherichia coli* flagellum biogenesis and motility. *Nucleic acids research*, 40(21), 11073-11085.

[4] Valdivia, R. H., & Falkow, S. (1996). Bacterial genetics by flow cytometry: rapid isolation of *Salmonella typhimurium* acid‐inducible promoters by differential fluorescence induction. *Molecular microbiology*, 22(2), 367-378.
